# 3D Electrospun Synthetic Extracellular Matrix for Tissue Regeneration

**DOI:** 10.1002/smsc.202100003

**Published:** 2021-05-25

**Authors:** Yingchun Su, Mette Steen Toftdal, Alice Le Friec, Mingdong Dong, Xiaojun Han, Menglin Chen

**Affiliations:** ^1^ State Key Laboratory of Urban Water Resource and Environment School of Chemistry and Chemical Engineering Harbin Institute of Technology Harbin 150001 China; ^2^ Department of Biological and Chemical Engineering Aarhus University DK-8000 Aarhus C Denmark; ^3^ Interdisciplinary Nanoscience Center (iNANO) Aarhus University DK-8000 Aarhus C Denmark; ^4^ Stem Cell Delivery and Pharmacology Novo Nordisk A/S DK-2760 Måløv Denmark

**Keywords:** native extracellular matrices, tissue regeneration, tissue engineering, 3D electrospun structures

## Abstract

Electrospinning is considered the most versatile micro‐/nanofiber fabrication technology. The electrospun fibers hold high surface area, desired mechanical properties, controlled topography, as well as the ease of biochemical functionalization. The 3D electrospun fibrous structures closely mimic the hierarchical architecture and fibrous features of the extracellular matrices (ECM), which greatly contribute to biomaterials design to stimulate tissue regeneration. Herein, the recent advances in electrospinning technology for 3D production of ECM‐mimicking biomaterial scaffolds are systematically summarized and the applications in neural, cardiac, bone, skin, and vascularized tissue regeneration are thoroughly discussed. Challenges and future scopes related to each field of tissue regeneration are discussed after each subsection. A few examples of liver, kidney, esophageal tissue engineering are also discussed. Finally, the key challenge in the cost‐effective upscale of the electrospinning technique to mature and prevalent industrial applications is outlined. Herein, a systematic, thorough summary of the recent evolutions in electrospinning and its emerging applications for a broad range of tissue regenerations is provided.

## Graphical abstract

Electrospinning as a versatile technology allows innovative advancement to fabricate 3D fibrous scaffolds that recapitulate the hierarchical architecture and biological features of the extracellular matrices. The emerging applications of these scaffolds in neural, cardiac, bone, skin, vascular, liver, kidney, and esophageal tissue regeneration are reviewed.

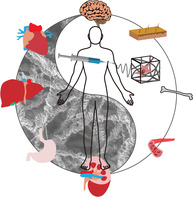

## 1 Introduction

Tissue regeneration is a rapidly evolving and interdisciplinary field at the intersection of life science, biology, material science, and engineering. Centrally, it involves functional cell‐free or cell‐laden constructs or biomaterial scaffolds that are fabricated utilizing various strategies.^[^ ^1^ ^]^

Cell‐free biomaterial scaffolds implanted directly at the sites of injury may mechanically support local cells to promote local tissue repair.^[^ ^2^ ^]^ Furthermore, they can be functionalized both on the surface or in the interior with bioactive materials to stimulate regeneration of functional tissues.^[^ ^3^ ^]^ In cell therapy applications, cell‐laden constructs may enhance both survival and differentiation of the therapeutic cells compared with cell transplantation alone. The grafted cells may then ideally replace lost tissue and/or exert beneficial effects on the host tissue.^[^ ^4^ ^]^ Among different biomaterial constructs, 3D fibrous scaffolds recreate a 3D microenvironment, which closely resembles the native extracellular matrix (ECM), including both structural and biochemical properties that guide cell survival and differentiation.^[^ ^5^ ^]^

Scaffolds with fibrous networks possess unique characteristics, including sufficiently high interconnected porosity, high specific surface area, tunable mechanical properties, as well as optimal morphological features. The high porosity of the fibrous scaffolds facilitates mass transfer for effective nutrient supply, oxygen diffusion, metabolic waste removal, and enhancement of intercellular communications, consequently allowing high cell viability and function throughout the entire scaffold.^[^ ^6^ ^]^ In addition, compared with 2D culture, the 3D cell culture provides a more realistic biochemical and biomechanical microenvironment,^[^ ^7^ ^]^ creating an optimal environment for cell migration, proliferation, and differentiation. Hence, nanofibrous scaffolds with appropriate biomechanical properties are highly suitable for tissue engineering.^[^ ^8^ ^]^ Electrospinning, as a relatively simple and versatile fiber preparation technique, has been developed to create fiber‐based constructs in combination with cells, bioactive molecules, proteins, and biocompatible nanomaterials.^[^ ^9^ ^]^

**Figure 1:**
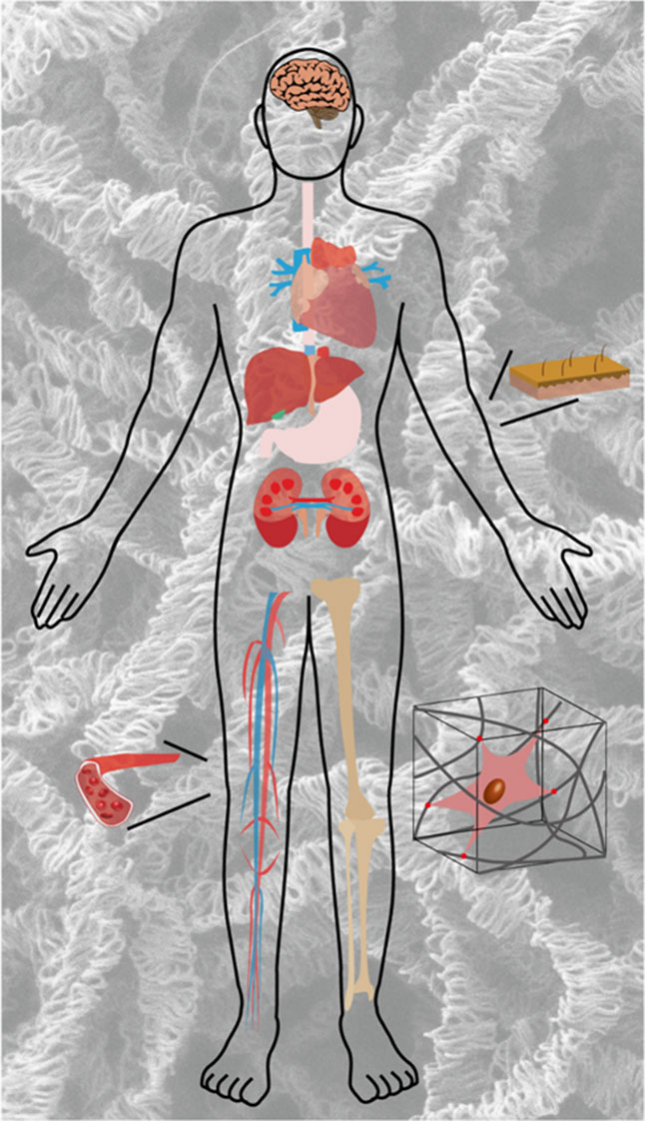
3D electrospun ECM scaffolds that made a significant contribution to various tissue/organ regenerations.

Many studies have demonstrated that the electrospinning technology has the potential for significant progress within the field of tissue regeneration. Chen et al.^[^ ^10^ ^]^ summarized the methods for producing electrospun 1D nanofiber bundles, 2D nanofiber membranes, and 3D nanofiber scaffolds and indicate the possible combinations of electrospinning with 3D printing, flexible electrodes, and microfluidics for biomedical applications. Ogueri et al.^[^ ^11^ ^]^ reviewed electrospinning in matrix‐based regenerative engineering, focusing mainly on musculoskeletal tissues. Sun et al.^[^ ^12^ ^]^ discussed electrospinning techniques for fabricating 3D nanostructures, and He et al.^[^ ^13^ ^]^ summarized electrohydrodynamic‐based bioprinting techniques, including near‐field solution electrospinning, melt electrowriting, and electrospray cell printing. There are recent reports of advances in tissue regeneration of bone,^[^ ^14^ ^]^ skin,^[^ ^15^ ^]^ vascular,^[^ ^16^ ^]^ and cardiac tissue.^[^ ^17^ ^]^ However, comparatively few reviews extensively discuss in‐depth fabrication processes together with bioengineering of diverse tissues. This review aims to update and summarize the most recent advancements in electrospinning technique toward fabricating 3D scaffolds, while also thoroughly reviewing emerging applications in neural, cardiac, bone, skin, vascular, liver, kidney, and esophageal tissue regeneration (**Figure** **1**). After discussing the breadth and depth of electrospun fiber biomaterials, we outline the key challenges and future perspectives of electrospinning for tissue regeneration.

## 2 Advancement of Electrospinning in 3D Production

**Figure 2:**
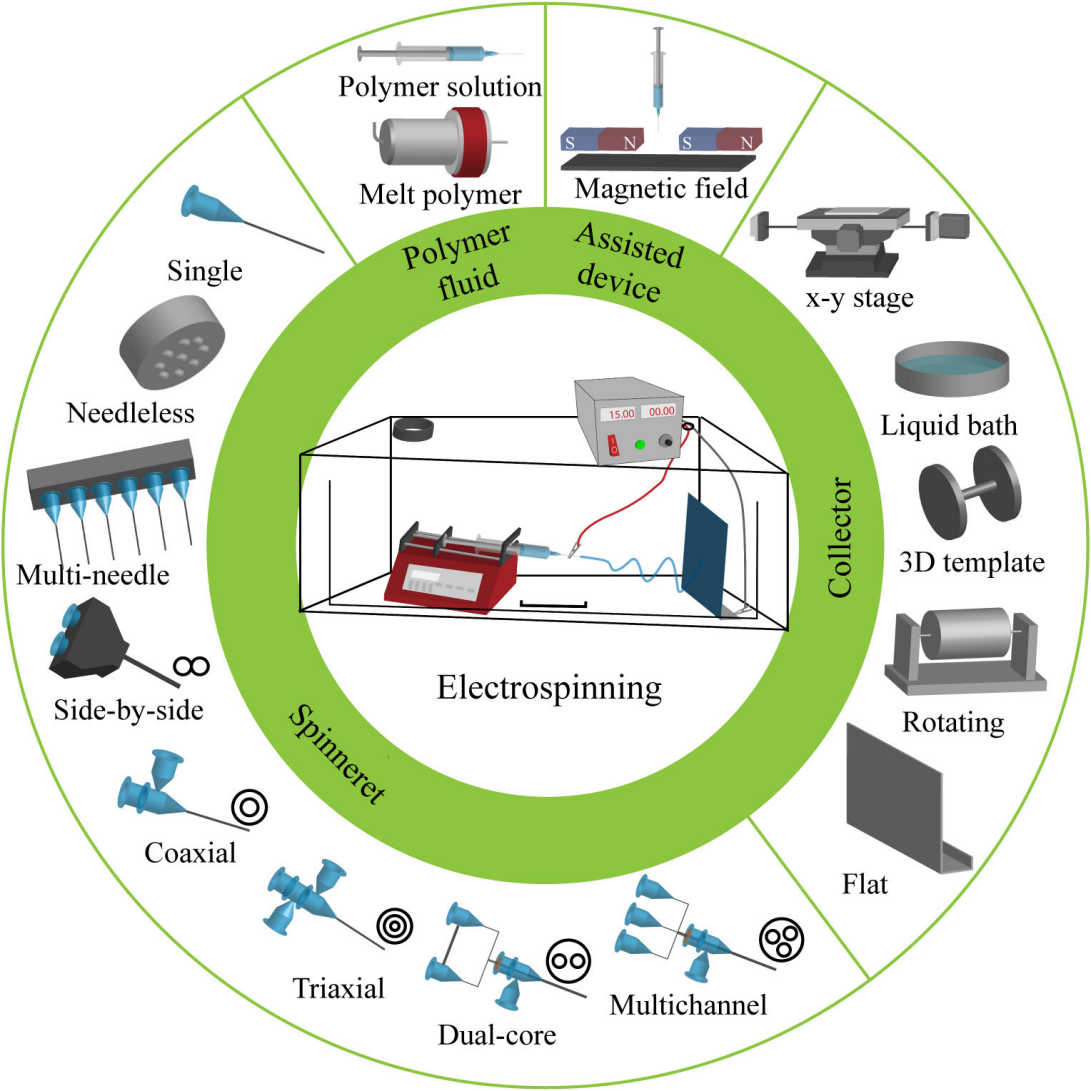
Schematic overview of a typical electrospinning device with the modified configurations utilized in different electrospinning processes.

Electrospinning is a highly versatile scaffold fabrication technique that combines an electric field with spinning to draw polymer solutions into micro‐ or nanofibers. The typical electrospinning setup is composed of four essential elements: 1) a syringe pump with a syringe that supports a stable flow of polymeric fluid through a flat‐tipped stainless‐steel needle to the initial jet point; 2) a high‐voltage power supply that provides a robust electrostatic force drawing the polymeric solutions into polymer jets; 3) a fume hood that can remove solvent evaporation from polymer jets and accelerate the solidification process during spinning; and 4) a metal collector plate for collecting the fibers. In the most commonly used electrospinning configurations, the stainless‐steel needle is connected with high voltage, and the collector is grounded. Some modified electrospinning devices use an opposite connection or a dual electrode with both positive and negative voltage. To further improve the electrospinning process, modifications of the spinneret/collector and addition of an assisted magnetic/electric field have been proposed in the literature. A graphical illustration of a simple electrospinning device and different modified electrospinning configurations are shown in **Figure** **2**.

Conventional electrospinning is known for fabricating densely packed submicrometer fibers. Simply increasing the electrospinning time is the easiest way to obtain scaffolds with a certain thickness. However several hours of electrospinning only increases the scaffold thickness by ≈0.14 mm,^[^ ^18^ ^]^ due to the loss of electrostatic force after insulating the collector with the collected fibers.

**Figure 3:**
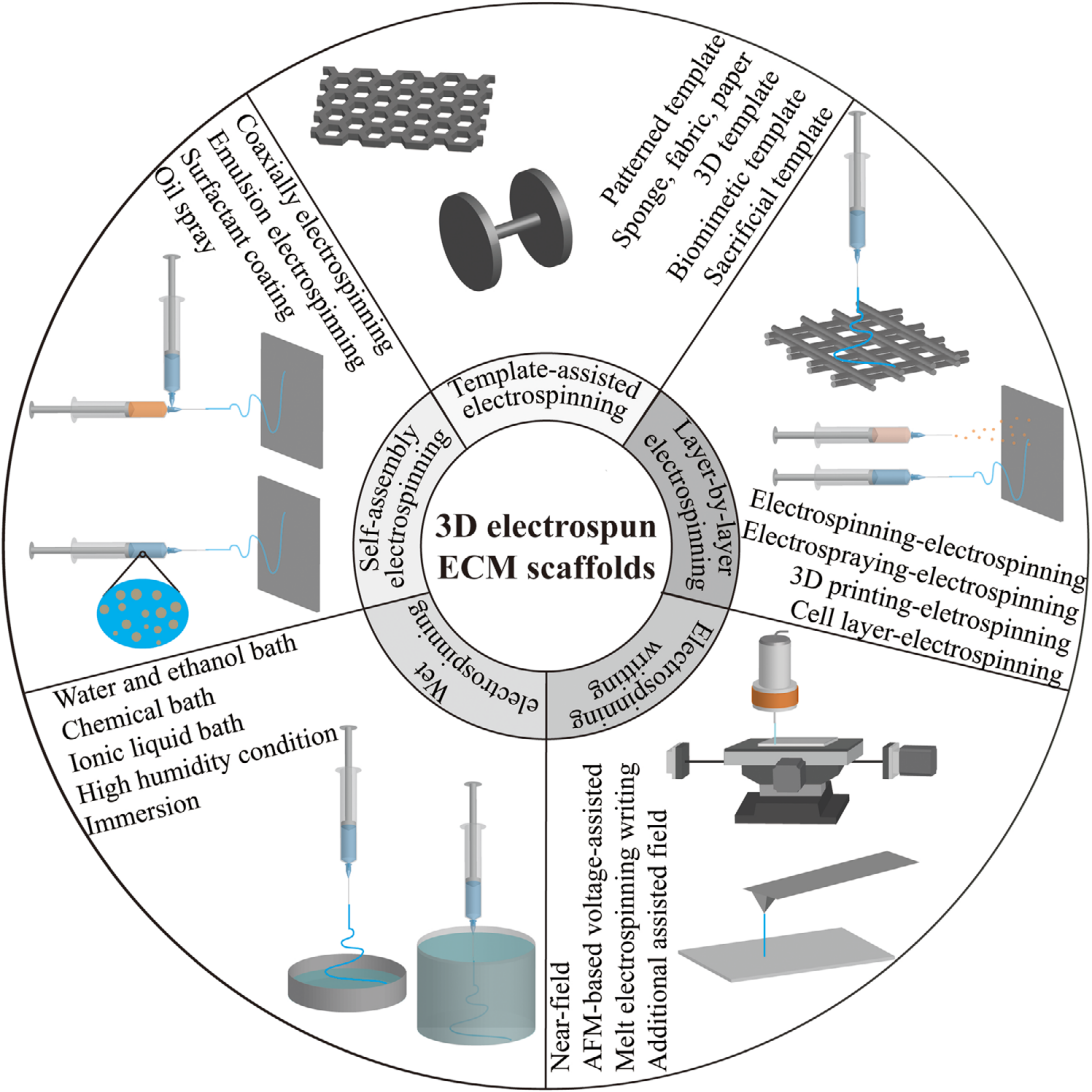
A summary of different electrospinning 3D production strategies.

Recently, 3D production of electrospun scaffolds has been attempted through the advancement of the electrospinning technology, shown in **Figure** **3**, including five major categories: 1) template‐assisted electrospinning; 2) self‐assembly electrospinning; 3) liquid‐assisted or wet electrospinning; 4) electrospinning writing; and 5) layer‐by‐layer electrospinning.

Template‐assisted electrospinning is one of the derived electrospinning techniques, which is done by modifying the shape of the collector. Using an insulated poly(methyl methacrylate) (PMMA) mask on the collecting copper plate to focus the collection of fibers, it is possible to produce scaffolds with 3 mm thickness in a relatively short time.^[^ ^19^ ^]^ The architecture of the fiber collector plates may be modified as well, to yield a template, which makes it possible to create customized 3D structures. Template‐assisted collectors are usually designed using either a computer‐aided design (CAD) program^[^ ^20^ ^]^ or conventional textiles.^[^ ^21^ ^]^ Many shapes such as honeycomb‐like,^[^ ^22^ ^]^ helical spring,^[^ ^23^ ^]^ metal pin,^[^ ^24^ ^]^ and micropatterned structures^[^ ^25^ ^]^ have been designed for various purposes. Furthermore, some biomimetic collectors, including auricle‐shaped^[^ ^26^ ^]^ and vascular‐like^[^ ^27^ ^]^ collectors, have been designed to be used for unique biomimetic tissue applications. The template itself may also be added to or produced in the process of electrospinning. For instance, microice crystals formed during low‐temperature electrospinning under high humidity were found to act as removable pore templates between the fibers. This resulted in enlarged distance between fibers, producing a 3D loosely packed architecture.^[^ ^28^ ^]^ Similarly, Park et al. proposed a method based on NaCl templates.^[^ ^29^ ^]^ Here they found that NaCl crystals increased the pore size of the electrospun fibers, improving cell proliferation within the scaffold. Template‐assisted electrospinning has furthermore been used in combination with microfluidic devices to improve the cell culture conditions under continuous flow.^[^ ^30^ ^]^

Alternatively, self‐assembly electrospinning can be used to increase the scaffold thickness and obtain 3D structures. Due to fast solidification from the polymer jets and the electrostatic attraction between the fibers on the grounded collector and the charged jets, the scaffold structure may grow toward the charged jets, thereby forming 3D structures with a thickness reaching more than 10 cm.^[^ ^31^ ^]^ Honeycomb‐patterned structures fall into the self‐assembly electrospinning category due to the electrostatic repulsion between adjacent fibers.^[^ ^32^ ^]^ This type of structural network has been intensively investigated due to the similarity to the osteon structure.^[^ ^33^ ^]^ The choice of polymer solution (polymer solutes and solvents) is the dominant effective factor when modifying the scaffold thickness in self‐assembly electrospinning. Other parameters, such as applied voltage, polymer concentration, and flow rate, etc., have less effect. So far, polystyrene (PS),^[^ ^31^ ^]^ polyacrylonitrile (PAN), poly(vinyl alcohol) (PVA), polyethylene oxide (PEO),^[^ ^32^ ^]^ and their hybrids^[^ ^34^ ^]^ reportedly exhibit self‐assembly properties during electrospinning. It is worth mentioning that surfactant coating (Beycostat A B09)^[^ ^35^ ^]^ and emulsion^[^ ^36^ ^]^ likewise promote the self‐assembly electrospinning process. As the electrostatic interactions and solidification rate are factors difficult to quantify, the optimization of self‐assembly electrospinning is still under investigation.

Generally, the collector type is a significant element in electrospinning and makes a great impact on the surface topography and the 3D geometry of the scaffolds. One type of collector is a coagulation bath in a metal container, which leads to liquid‐assisted collection, also called wet electrospinning.^[^ ^37^ ^]^ Ethanol,^[^ ^38^ ^]^ mixed ethanol/water solution,^[^ ^39^ ^]^ hexane,^[^ ^40^ ^]^ or subcritical CO_2_ fluid^[^ ^41^ ^]^ have been used as coagulating agents. In addition, some of the chemicals in the solutions may induce crosslinking^[^ ^42^ ^]^ or functional surface coating on the fibers.[^38^, ^43^] An important aspect to consider for the collector bath is the surface tension of the collector solution. Solutions with low surface tension for target fibers create loosely packed 3D fibrous scaffolds. Conversely, solutions with high surface tension resemble a traditional collector plate, where the fibers are collected on top of the solution, resulting in densely packed fibers. It is noteworthy that electrolyte solutions (e.g., KCl solutions) can act as a ground collector that combines the functions of the metal container and the coagulation bath into one. This yields a more effective way to fabricate free‐standing, complex nanofiber architectures according to the designed patterns.^[^ ^44^ ^]^ High humidity^[^ ^45^ ^]^ and immersions^[^ ^46^ ^]^ are variations of the wet electrospinning technique. Wet electrospinning has further been combined with a rotating collector,[^39^, ^42^] layer‐by‐layer^[^ ^47^ ^]^ electrospinning, and template electrospinning^[^ ^44^ ^]^ for various 3D scaffolds. In addition, post‐treatment with immersion and freeze drying may be helpful in transforming dense electrospinning membranes into loosely packed 3D structures.^[^ ^48^ ^]^

Further improvements have been made to the traditional electrospinning technique, such as applying an *x–y* translational motion collector, similar to the one used in standard 3D printing.^[^ ^49^ ^]^ Utilizing the jetting initiated prior to the onset of bending stability, high‐precision and direct‐write deposition of microfibers have been achieved by decreasing the electrospinning distance, a technique called near‐field electrospinning (NFE). As an extension to NFE, the atomic force microscope (AFM) system (AFM‐based voltage‐assisted electrospinning) allows fabrication of nanofibers in a controllable fashion via polymer deposition from an AFM probe.^[^ ^50^ ^]^ Initially, NFE was mainly applied for polymers dissolved in solvents.^[^ ^51^ ^]^ As evaporation of solvents may affect the precision of the fiber deposition,^[^ ^52^ ^]^ solvent‐free electrospinning was introduced to the field of NFE in 2008, creating the emerging melt electrospinning writing (MEW) technology.^[^ ^53^ ^]^ Although MEW makes it possible to achieve high‐resolution nanostructures with tunable coil densities^[^ ^54^ ^]^ and precise deposition of single fibers,^[^ ^49^ ^]^ it cannot achieve wide‐range regulation in height. This challenge must be overcome before MEW is fully applicable in the construction of artificial organs. Combining MEW with template‐assisted collectors could be one way of addressing this limitation.^[^ ^55^ ^]^

Besides using NFE and MEW, controlled deposition of fibers has been achieved using an additional magnetic/electric field to standard electrospinning. Using a cylindrical side‐wall electrode and a sharp‐pin ground electrode, the electrospinning jet was focused and a patterned nanofibrous mat was fabricated.^[^ ^56^ ^]^ However, until now, aligned architecture and tunable deposition area have only been achieved for bundles of ﬁbers.^[^ ^57^ ^]^ Inspired by MEW, polymer melts may possibly be utilized in future field‐assisted electrospinning techniques to potentially yield precise control of single‐fiber deposition.^[^ ^49^, ^58^ ^]^ Field‐assisted electrospinning could become the next‐generation leading electrospinning technique for single‐fiber deposition.

Last but not the least, layer‐by‐layer electrospinning may be the most flexible strategy. It combines not only different electrospinning methods,^[^ ^59^ ^]^ but also other technologies, such as cell layer and electrospinning layer,^[^ ^60^ ^]^ 3D printing layer and electrospinning layer,^[^ ^61^ ^]^ and electrospraying layer and electrospinning layer.^[^ ^62^ ^]^

**Table** **1** shows the six electrospinning techniques covered in this Review with their pertinent features and limitations. It is foreseen in the future that even more techniques could be incorporated into the versatile electrospinning platform.

**Table 1:** Categorized electrospinning 3D production strategies along with their advantages and disadvantages

Categorized techniques	Max thickness	Typical materials	Features and characteristics	Advantages	Disadvantages
Increasing spinning time	≈140 μm ^[^ ^18^ ^]^	PCL, PMMA, polyethylene terephthalate, poly(L‐lactide), silk fibroin (SF)	Traditional electrospinning	The broad range of materials; no additional setup; the simplest method	Thicknesses limited; long spinning time
Template‐assisted electrospinning	Centimeter scale,^[^ ^167^ ^]^ depending on the template	PCL, poly(vinylidene fluoride), poly(ester‐urethane), poly(lactic acid*‐co‐*glycolic acid), polylactide, PEO, collagen, polyaniline (PANI), PS	3D template‐assisted collection; patterned collector with accurately controlled geometries	The broad range of materials; direct fabricating; good controllability (porosity, fiber organization, and geometry)	Depend on the shape and geometry of collector; customized collector
Self‐assembly electrospinning	10 cm for 30 min^[^ ^31^ ^]^	PCL, poly(L‐lactide), PS, poly(vinylpyrrolidone), PEO, pellethane, poly(lactic acid) (PLA), poly (carboxybetaine*‐co‐*methyl methacrylate) co‐polymer, poly(vinylidene fluoride*‐co‐*trifluoroethylene)	Honeycomb‐like self‐organization; fast solidification rate; electrostatic interaction	Highly porous fibrous sponges; no additional setup; mechanical stability	Limited materials; long spinning time
Wet electrospinning	2.3 cm for 40 min^[^ ^168^ ^]^	PCL, SF, poly(lactic*‐co‐*glycolic acid) (PLGA), polyamide, pullulan (PUL), cellulose acetate (CA), cellulose	The lower surface tension of solution; liquid‐assisted collection	Cotton‐like architectures; crosslinking or coating process in a bath; simple setup	Additional solution bath
Electrospinning writing	7.1 mm,^[^ ^58^ ^]^ depending on the number of layers and the thickness of one layer	PCL, poly(urea‐siloxane), poly‐hydroxymethylglycolide*‐co‐*ε‐caprolactone, poly(2‐ethyl‐2‐oxazoline), polypropylene	The convergence of 3D printing stage and electrospinning; NFE; layer‐by‐layer deposition	Highly ordered scaffold; reproducible print; tunable width and depth; single‐fiber controllability	Limited materials and thickness; high‐temperature process
Layer‐by‐layer electrospinning	2.8 mm,^[^ ^169^ ^]^ depending on the thickness of the other layer	PCL, chitosan (CS), poly(L‐lactide), nylon 6	Postprocessing method; multilayer electrospinning	Broad range of materials; controllable layer number; different designed layers	Lack continuity; the long‐distance gap between adjacent layers

## 3 Tissue Regeneration Based on 3D Electrospun Scaffolds

3D electrospun scaffolds can help fabricate highly porous structures with biomaterials mimicking the natural 3D extracellular microenvironment for improved cell adhesion, proliferation, and differentiation.^[^ ^54^, ^63^ ^]^ 3D electrospun scaffolds display strong potential in tissue regeneration through their biochemical cues from natural, synthetic, or hybrid biomaterial selections and physical cues from the mechanical, topographical, and geometrical features of the fiber constructs.[^38^, ^64^] Most recent studies have focused on producing 3D bioactive and biocompatible scaffolds with potential applications in fields such as neural, cardiac tissue, and bone tissue regeneration.[^63^, ^65^] The following sections seek to give an in‐depth description of the role of electrospinning as a tool in tissue regeneration applications.

### 3.1 Neural Tissue Regeneration

Millions of people worldwide suffer from peripheral nerve injuries and central nervous system (CNS) damage produced by stroke, traumatic brain injury, neurodegenerative diseases, spinal cord injuries. Though the CNS is highly plastic and to some degree capable of self‐regeneration, lost neural tissue is often not fully replaced. Therefore, much hope for the treatment of CNS injury lies in tissue engineering approaches. As the fibers can provide physical guidance and topical stimulation in the repair process of nerves, and fibrous nerve conduits can be introduced at lesion sites by implantation, many in vitro investigations have focused on optimizing electrospun fibrous scaffolds to provide a suitable microenvironment for neural growth.^[^ ^66^ ^]^

**Figure 4:**
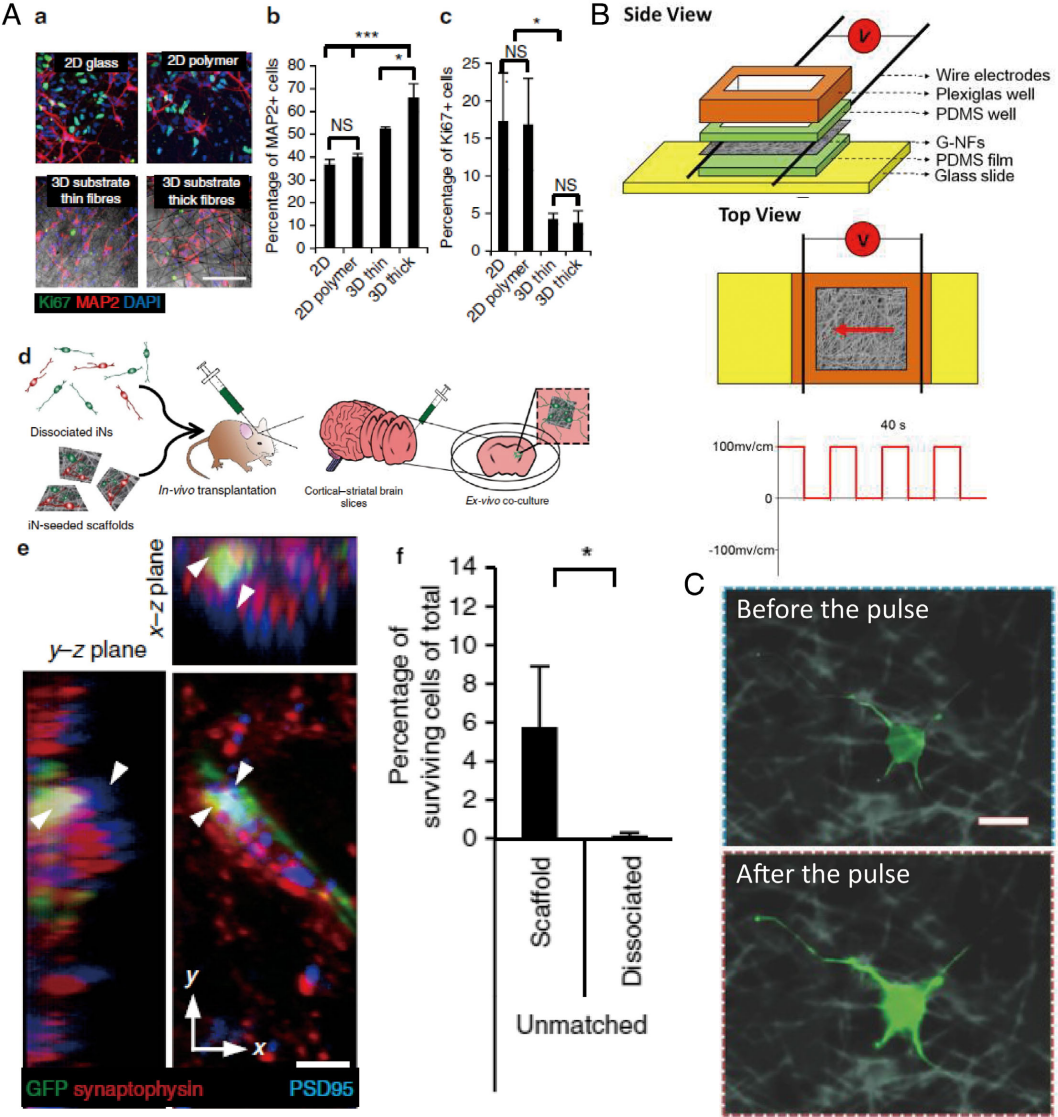
A) a) Immunocytochemistry for Ki67 and MAP2 marker expression. Quantification reveals of maturation assessed by b) MAP2 expression and c) neuronal selection assessed by Ki67 expression in 2D and 3D substrates. Scale bar, 100 μm. *n* = 3, *P < 0.05, ***P < 0.001. d) iN‐seeded scaffolds were injected into mouse striatum. e) Postsynaptic density protein 95 (PSD‐95, blue, indicated using downward‐pointing arrows) was detected adjacent to transplanted green fluorescent protein‐labeled iN neurite terminals (green), which colocalized with synaptophysin (red), resulting in yellow regions indicated with upward‐pointing arrows, suggestive of synaptic integration with host tissue. Scale bar, 5 μm. f) iNs supported by scaffold support survival in vivo. Reproduced under the terms of the CC‐BY 4.0 license.^[63c]^ Copyright 2016, The Authors, published by Springer Nature. B) Schematic representation of the artificial device used for ES. C) The ﬂuorescence images of the cells preincubated with Fluo‐4AM (membrane‐permeable and Ca^2+^‐dependent dye) on graphene electrically conductive nanofibers before and after the pulse. Green: Ca^2+^. Scale bar is 10 μm. B,C) Reproduced with permission.^[^ ^75^ ^]^ Copyright 2015, Wiley‐VCH.

Combining the 3D structures with bioactive cues, inspired by the composition of the ECM, is one way to improve scaffold performance. For example, covalently bonded laminin^[66a]^ and immobilized brain‐derived neurotrophic factor^[^ ^67^ ^]^ combined with electrospun scaffolds were found to promote neurite extension. In addition, basement membrane extract used as a coating material was found to yield increased production of bioactive nerve growth factor (NGF) and vascular endothelial growth factor (VEGF) by rat embryonic stem cells.^[^ ^68^ ^]^ A PCL/CS/polypyrrole (PPy) nanofibrous scaffold significantly increased cell proliferation up to 356% in comparison with pure PCL.^[^ ^69^ ^]^ A key component of the ECM, glycosaminoglycan (GAG), was coated onto the surface of electrospun scaffolds and enhanced Schwann cell activity.^[^ ^70^ ^]^ GAG mimetic containing cellulose sulfate (CelS) and partially sulfated cellulose sulfate (pCelS) was introduced into gelatin scaffolds, an approach that increased NGF binding to the scaffold and neurite extension of dorsal root ganglion neurons.^[^ ^71^ ^]^ To sum up, 3D structures combined with bioactive cues have proven a fairly straightforward way of providing a suitable substrate for neural growth and maturation. Interestingly, these benefits have also been observed after transplantation in vivo. 3D fibrous architectures were found to selectively reduce the presence of residual proliferative‐induced pluripotent stem (iPS) cells (proliferation marker Ki67) and accelerate maturation in vitro (higher expression of microtubule‐associated protein 2, MAP2) (**Figure** **4**A a–c). These cell‐laden scaffolds were transferred into the mouse striatum in vivo (Figure 4A d). Three weeks after transplantation, postsynaptic density protein 95 (PSD95, depicted in blue, with downward‐pointing arrows) was detected adjacent or colocalized to transplanted iPS‐induced neuronal (iN) neurite terminals (Figure 4A e), indicating synaptic integration with the host tissue. In addition, these 3D electrospun scaffold‐supported neuronal networks were found to enhance survival and engraftment of neurons in the murine brain tissue after injection compared with injection of isolated cells (Figure 4A f).^[63c]^

As neurons are electroactive, conductive scaffolds recently received considerable attention in the field of neural tissue regeneration due to their potential in improving tissue prosthetics using electrical stimulation (ES) and bioelectronic signal transfer.^[^ ^72^ ^]^ In vitro, conductive materials have been shown to promote growth and differentiation of neural and neural‐like cells.^[^ ^73^ ^]^ For instance, application of ES using a triboelectric nanogenerator device significantly accelerated nongenetic neuronal conversion of mouse fibroblasts to iN cells.^[^ ^74^ ^]^ A schematic representation of an artificial device used for providing such ES is shown in Figure 4B.^[^ ^75^ ^]^ Highly conductive carbon nanomaterials, such as graphene or reduced graphene oxide (rGO), have gained significant interest in recent years,^[^ ^76^ ^]^ due to their remarkable properties such as high surface area, high mechanical strength, and ease of functionalization. Modified 0.5% rGO has been incorporated into poly(ester amide) and found to reduce film resistance by at least 10^4^ Ω sq^−1^.^[76b]^ rGO has been reported to have low dispersibility in different polymer matrices. To overcome this problem, Gou et al. increased the dispersibility of rGO polymer matrix by grafting trimethylene carbonate (TMC) oligomers onto rGO.^[^ ^77^ ^]^ With electrical pulses, rGO has been reported to accelerate nerve growth and increase expression of βIII‐tubulin, MAP2, and nestin.[^72^, ^75^] This signiﬁcant increase in neural marker expression was due to the inﬂux of Ca^2+^ induced by a depolarizing current, which consequently was found to activate the calmodulin kinases to elicit neurites outgrowth and development. Calcium influx can be assessed by the relative ﬂuorescence intensity change during a pulse period when preincubated with Ca^2+^‐dependent dye (Figure 4C).^[^ ^75^ ^]^ Even conductive materials without ES may be used to influence the cell behavior by enhancing transmission of electrical signals between cells. For example, graphene/polyelectrolyte 3D architecture enhanced neuron cell adhesion and neurite outgrowth.^[72a]^ Polyurethane(PU)/silk‐functionalized multiwalled carbon nanotube scaffolds^[^ ^78^ ^]^ likewise significantly improved expression of neural markers in vitro and increased axonal growth without any ES. Another approach to obtain artificial electrical active neural network systems involved coating metal such as gold onto the surface of electrospun fibers.^[^ ^79^ ^]^

Besides inorganic nanomaterials, promising conductive polymers have been under investigation. Loosely packed 3D conductive PAN/PPy electrospun nanofibers increased differentiation and migration of cortical cells after ES.^[^ ^80^ ^]^ Adding PPy to the polymer blend was found to enhance the growth of rat pheochromocytoma 12 (PC12) neural‐like cells by 59% by increasing the electroactivity of nanofibrous scaffold.^[^ ^69^ ^]^ Electrically conductive PANI/PCL microfibers hold a conductivity above that of biological fluids (7.7 × 10^−2^ S cm^−1^ vs 1.0 × 10^−2^ S cm^−1^), making these ideal candidates for in vitro neural differentiation studies under ES.^[^ ^81^ ^]^ An iron‐containing porphyrin, hemin, has likewise been doped to confer conductivity in serum albumin‐based scaffolds (≈2 mS cm^−1^).^[^ ^82^ ^]^ Not only direct ES is useful in neural tissue regeneration, magnetic stimulation and light stimulation have likewise proven their potential. Magnetic fields generated from magnetostrictive ﬁller graphene oxide (GO)/CoFe_2_O_4_ nanoparticles together with piezoelectric polymer polyvinylidene difluoride (PVDF) induced mesenchymal stem cells (MSCs) growth and differentiation into neural cells without using chemical induction differentiation media.^[^ ^83^ ^]^ Light stimulation of inorganic/organic semiconductive materials, such as g‐C_3_N_4_/GO^[^ ^84^ ^]^ and poly(3‐hexylthiophene),^[^ ^85^ ^]^ has been shown to trigger optoelectronic conversion, thus providing a novel means of ES. External electrical stimuli of 3D conductive scaffolds open the possibility of influencing cellular behavior, which may potentially benefit the therapeutic applications.

**Figure 5:**
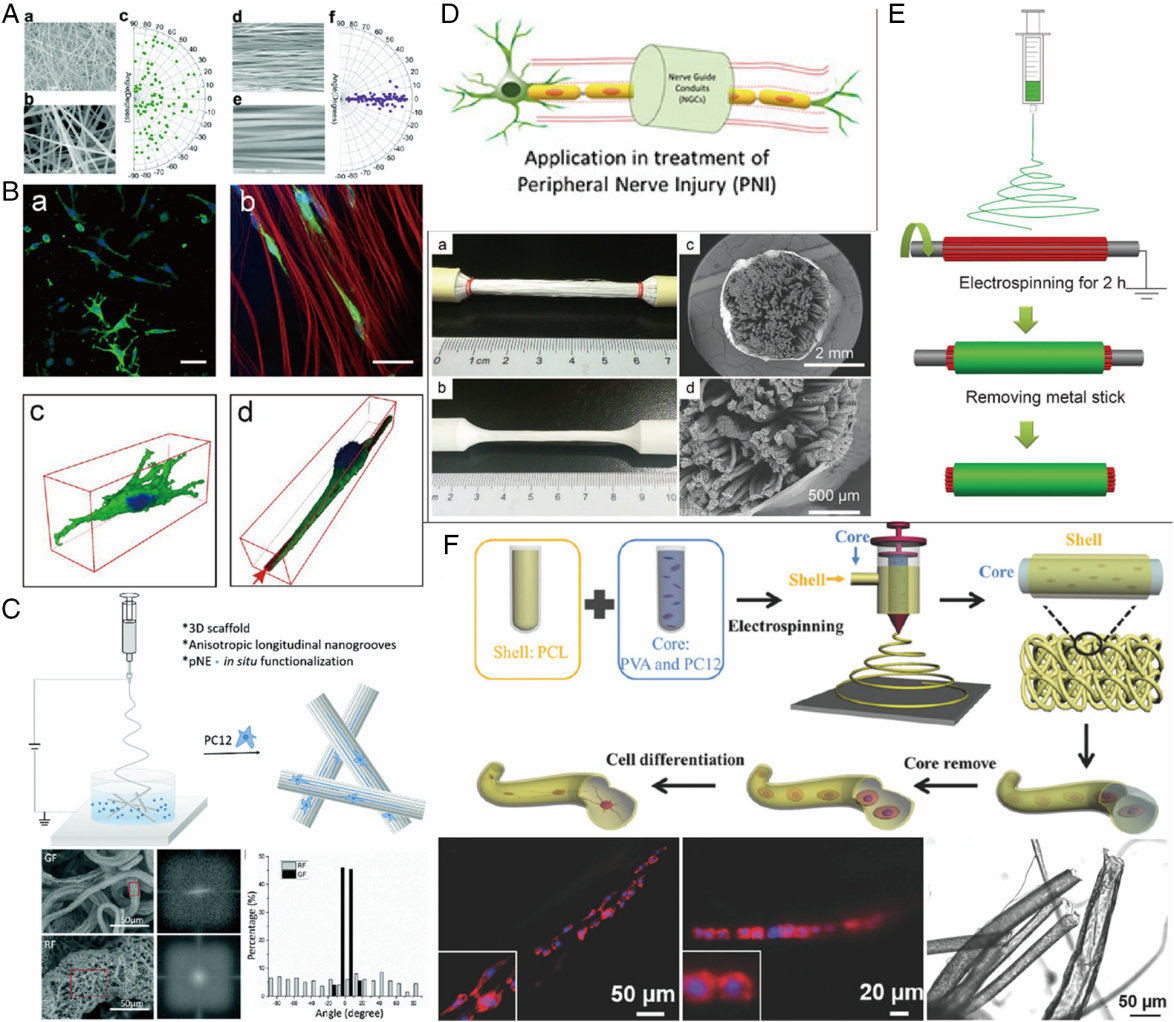
A) Scanning electron microscopy (SEM) images at different magnifications and corresponding angle distributions for a−c) random and d−f) aligned polyphenylene sulfone electrospun nanofibers, respectively. Reproduced with permission.^[86a]^ Copyright 2018, Royal Society of Chemistry. B) PCL fibers affect cell morphology. Scale bar is 50 μm. Reproduced with permission.^[86b]^ Copyright 2013, Wiley Periodicals, Inc. C) The fabrication of bioadhesive anisotropic nanogrooved fibrous scaffold for 3D neural culture. SEM images with Fourier transform analysis (FFT) analysis of the selected red zone and groove angle distribution of grooved fiber (GF) and RF. Reproduced with permission.^[38b]^ Copyright 2019, Royal Society of Chemistry. D) The schematic of nerve guide conduits. Reproduced under the terms of the CC‐BY 4.0 license.^[^ ^90^ ^]^ Copyright 2019, The Authors, published by Elsevier. E) Incorporation of the nanofiber yarn into the conduit. Reproduced with permission.^[^ ^91^ ^]^ Copyright 2015, Royal Society of Chemistry. F) The preparation of PCL/PVA coaxial microfibers with PC12 cell embedded. Reproduced with permission.^[92a]^ Copyright 2018, Wiley‐VCH.

Equally important, neural cells in the native tissues tend to align in certain directions and a tissue's function is tied to its structure. Oriented electrospun fibers with aligned topographies can support growth and migration along specific directions (**Figure** **5**A,B).^[^ ^86^ ^]^ Aligned scaffolds reportedly provide more contact guidance for neural differentiation, resulting in enhanced nestin and βIII‐tubulin expression compared with random fibers (RFs).^[^ ^87^ ^]^ Likewise, PEO/PCL/poly(norepinephrine) microfibers with aligned nanogrooved channels (GF) have been found to significantly promote neurite extension in 3D (Figure 5C).^[38b]^ Besides fiber structure (random or aligned), fiber diameter and density also have an effect on cell growth and neurite extension. Daud et al. found that 8 μm fiber diameter promoted neurite outgrowth for neuronal cells alone, and 1 μm fibers were found to be superior in terms of neurite outgrowth in the tested coculturing system with Schwann cells.^[^ ^88^ ^]^ Therefore, under different conditions, the diameter should be optimized. High fiber density may result in neurite path alteration and reduce neurite linearity.^[^ ^89^ ^]^ Scaffolds that allow aligned growth of neurons could hold particular promise for the repair of injured nerve fibers by bridging the gap between the two stumps of a severed nerve. So‐called nerve guidance conduits (NGCs) are tubular structures designed to guide the regeneration of nerve cells, as shown in Figure 5D.^[^ ^90^ ^]^ The highly aligned nanofibers in the nerve conduits served as guidance for axon spreading and cell migration of Schwann cells in vitro (Figure 5E).^[^ ^91^ ^]^ PC12 cells have been directly encapsulated in hollow coaxial fibers to form living 3D‐connected neuronal networks (Figure 5F), where excellent alignment of the cells was observed along the directions of the microfibers.^[^ ^92^ ^]^ In vivo, the oriented 3D fiber structure facilitates not only cell migration and guidance of the axonal extension, but also provides increased flexibility and resistance to deformation in a rat model of spinal cord injury.^[^ ^93^ ^]^ In another work, aligned fibers within NGCs enhanced the bridging of the injured sciatic nerve in rats.^[^ ^94^ ^]^ Interestingly, randomly oriented nanofibers could be used as a coating layer to strengthen the mechanical properties of the aligned fibers.^[^ ^95^ ^]^

**Figure 6:**
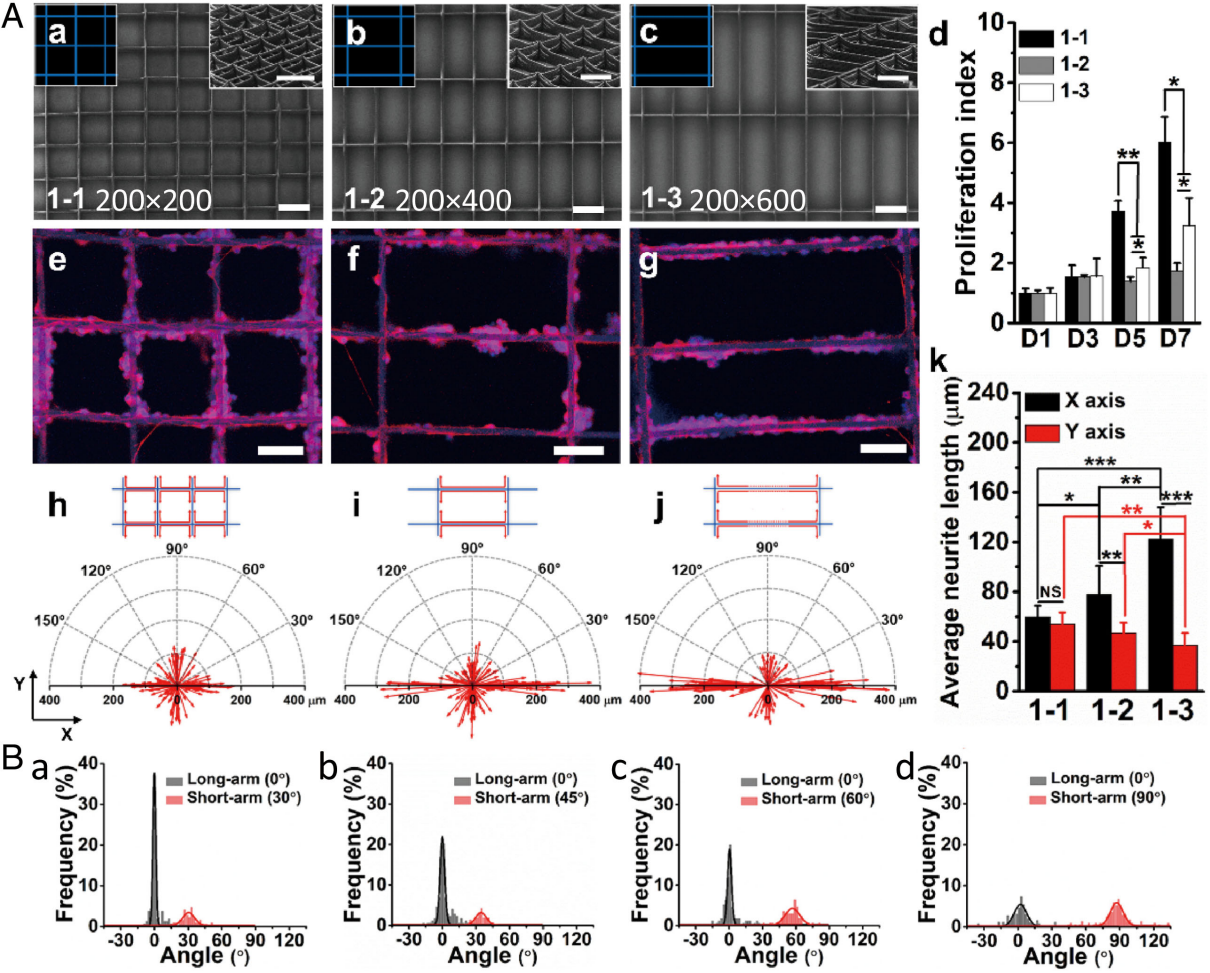
Guiding effect of 3D grid PCL architectures on neurite outgrowth. A) SEM images (a–c, scale bars: 200 μm) of melt electrowritten PCL scaffolds and immunofluorescence images (d–f, scale bars: 100 μm) of PC12 cells after 7 days differentiation. Cells were stained with class III β‐tubulin (TUJ1, red) and Hoechst (blue). Compass plot of neurite length/orientation (h–j) of cells cultured on the three scaffolds. d) Cell proliferation index, and k) average neurite length analysis of cells cultured on the three scaffolds. Statistical data are denoted as *p < 0.05, **p < 0.005, ***p < 0.001. B) Histograms on the orientations (a–d) of neurite extensions on these patterned scaffolds (1–2) (30°, 45°, 60°, and 90°). A,B) Reproduced with permission.^[84b]^ Copyright 2020, Elsevier.

The strength of electrospinning is that it allows the creation of a wide diversity of scaffold morphologies. Zhang et al.^[84b]^ developed three different anisotropic gridded PCL scaffolds with varying spacings between the grid patterns (**Figure** **6**A). Tailoring of the fiber spacing ratio between two arms of the grid patterns was found to affect the neurite extension of the seeded PC12 cells (Figure 6A‐d). The largest fiber spacing (600 μm from 1–3 scaffold) was found to induce increased biased growth along the long arm direction (Figure 6Ak). Intersection angles were also an important parameter to control, as anisotropic neurite guidance was further strengthened when the intersection angles were reduced from 90° to 30° (Figure 6B).

Due to the significance of aligned fibrous topography and ES, scientists have tried to combine the two properties to investigate the potential additive effects. In vitro, the combination of aligned fiber morphology with electrical activation increased the expression of neuron‐specific cytoskeletal proteins and microtubule assembly of S42 Schwann cells in PC12 cells.^[^ ^96^ ^]^ The directional alignment of neurites in PCL/poly(acrylic acid) conductive scaffolds was likewise reported to enhance protein expression of βIII‐tubulin and neurofilament 200.^[^ ^90^ ^]^ Also, a rolled‐up MoS_2_−PVDF nanofiber film with good electrical conductivity and large surface area significantly increased cell attachment and proliferation.^[73a]^ Through a combination of bioactive cues, aligned topographies, conductive coating, and ES, CS/PPy‐coated poly(L‐lactic acid) (PLLA)/PCL fiber films with 100 mV of ES were found to enhance neural cell compatibility and neurite growth.^[^ ^97^ ^]^ In vivo, the implantation of aligned conductive electrospun scaffolds in rats after spinal cord injury improved functional recovery and electrical signals measured as motor‐evoked potentials.^[^ ^98^ ^]^ Thus, multicue scaffolds may hold great potential for application in neural tissue regeneration. The mechanisms underlying the beneficial effects of ES, however, require further exploration.

**Table** **2** shows the 3D electrospun scaffolds for neural tissue regeneration according to the functional elements, such as 3D architecture, bioactivity, aligned topography, conductivity, as well as stem cell sources used for validation. As the nerve cells are highly organized in the spinal cord, scaffold topography is likewise an important element in mimicking the native ECM. Electrical stimulation is a relatively new method in neural tissue regeneration and is proposed to mimic the natural cell−cell electrical communication and thereby stimulate cell growth and differentiation.

**Table 2:** 3D electrospun scaffolds for neural tissue regeneration

Functional elements	Polymer substrate	Scaffolds structures	Fabrication techniques	Functional outcomes	Cell type	Refs.
3D architecture	PCL	Random and aligned submicrometer fibers	Increasing spinning time	Upregulation of genes associated with healthy mature astrocytes phenotypes	Primary murine astrocytes	[170]
	PCL/poly‐L‐lysine	Low‐density and uncompressed nanofibers	Template‐assisted electrospinning	Promoted neuronal differentiation into 3D‐integrated networks, formation of inhibitory and excitatory synapses, and extensive neurite growth.	Human neural progenitor cell line	[171]
Bioactivity	PVA/CS	Random nanofibers	Increasing spinning time	Promoted differentiation into neuron‐like cells expressing MAP2 and βIII‐tubulin.	Human dental pulp stem cells	[172]
	PS/laminin	Random submicrometer fibers	Increasing spinning time	A 3D structure coupled with laminin isoforms increases the expression of stemness markers providing a model to study glioblastoma.	U251 human glioblastoma cells	[63a]
	CA/PLA/citalopram‐loaded gelatin nanocarriers	Random submicrometer fibers	Wet electrospinning; rolling up	Improved functional recovery after sciatic nerve injury in rats.	Primary rat Schwann cells	[173]
Aligned topography	Poly(L‐lactide*‐co‐*caprolactone)/PLLA	Nerve conduit tube (random nanofibers) with inner aligned nanofibers	Template‐assisted electrospinning	Increased proliferation and spreading of cells across the material, axons extended along the fibers.	Schwann cells	[91]
	PCL	Aligned microfibers	Increasing spinning time	Increased neurite outgrowth and cell migration of Schwann cells.	NG108‐15 neuronal cells and primary rat Schwann cells	[88]
Aligned topography and bioactivity	Photo‐crosslinked gelatin methacryloyl (GelMA)	Aligned micro‐/nanofibers	Increasing spinning time; rolling up	Promoted migration of neural stem cells and differentiation into neuronal cells. In vivo the scaffold inhibited glial scar formation and promoted angiogenesis.	Bone mesenchymal stem cells (BMSCs) and hippocampal neuronal cells	[93]
	PCL/PEO/poly(norepinephrine)	Nanogrooved microfibers	Wet electrospinning	Increased neurite extension.	PC12 cells	[38b]
	PCL/gelatin or laminin	Aligned nanofibers	Increasing spinning time; layer‐by‐layer method	Increased neurite outgrowth in controlled directions.	Enhanced green fluorescent protein (eGFP)‐expressing neuronal SH‐SY5Y human cell line	[66b]
Conductivity	Carbon	Patterned nanofibers	Template‐assisted electrospinning	Supported cell adhesion and survival	E18 rat cortical neurons	[174]
	MoS_2_−PVDF	MoS_2_ nanoflakes on electrospun PVDF nanofibrous matrices	Increasing spinning time; hydrothermal assembly	Offered good electrical conductivity and promoted NSC attachment, spreading, and differentiation.	Neural stem cells from rat embryonic cortex	[73a]
Conductivity and bioactivity	Graphene‐heparin/poly‐L‐lysine polyelectrolytes/PCL	Random and aligned nanofibers	Increasing spinning time	Passively conductive (can transmit applied electrical stimuli), supported neuron cell adhesion, and neurite outgrowth.	Primary cortical neurons from embryonic day 14 (E14) C57/B6 mice	[72a]
	PLGA/GO/methylene blue	Random nanofibers	Increasing spinning time	Culture on the scaffold activated the autophagy signaling pathway, resulting in reduced tau phosphorylation and protection from apoptosis.	Neural progenitor cell from E14 C57 mouse	[175]
	PPy/SF	Random SF nanofibers on aligned PPy/SF scaffolds	Layer‐by‐layer electrospinning	Enhanced cell adhesion, differentiation, and proliferation	Rat primary Schwann cells and mouse fibroblast cells (L929)	[73b]
Conductivity, aligned topography, and bioactivity	PCL/SF/carbon nanotubes/hydrogel	Aligned nanofiber yarn	Dry−wet electrospinning	Neurite extension along the fibers, protection of cell organization by a hydrogel shell.	PC12 cells and neonatal rat dorsal root ganglia cells	[64a]
Electrical stimulation	Graphene/poly(vinyl chloride)	Random nanofibers	Increasing spinning time	Increased growth of primary motor neurons in response to ES.	Spinal cords of E15 Sprague−Dawley rats	[75]
	100 mV cm^−1^ (40s) was conducted every other hour.					
	PAN/PPy	Random nanofibers	Wet electrospinning	Increased glial cell proliferation and neuron maturation in response to pulsed ES.	Rat cortical cells	[80]
	100 mV cm^−1^, 100 Hz pulsed electrical field for 4 h day^−1^.					
Electrical stimulation and aligned topography	PEO/poly (3,4‐ethylenedioxythiophene): polystyrenesulfonate	Random and aligned nanofibers	Increasing spinning time	Increased neurite outgrowth in response to ES.	PC12 cells	[176]
	100 mV cm^−1^ (100 ms) for 1 h day^−1^.					
Electrical stimulation and bioactivity	SF/rGO	Random and aligned nanofibers	Increasing spinning time	Increased cell survival and higher gene expression of βIII‐tubulin, MAP2, and nestin in response to ES	Human conjunctiva MSCs	[72b]
	115 V m^−1^, 100 Hz or 0.1 Hz for 10 min day^−1^					
Light stimulation	PCL/GO/g‐C_3_N_4_	Grid micropatterns	Electrospinning writing	Preferential neurite extension of PC12 cells along the long arm direction and reduced angle; further enhanced neurite extension under visible‐light stimulation.	PC12 cells	[84b]
Light stimulation and bioactivity	PCL/gelatin/ascorbic acid/GO/g‐C_3_N_4_	Random microfibers	Increasing spinning time	Accelerated neuronal differentiation; increased neurite outgrowth under visible‐light stimulation.	PC12 cells	[84a]

### 3.2 Cardiac Tissue Regeneration

Cardiovascular diseases are the leading course of death compared with any other disease, affecting millions of people globally and significantly decreasing their quality of life.^[^ ^99^ ^]^ The human heart has low regenerative properties, making this tissue very vulnerable to injuries such as myocardial infarction (MI). After MI, the injured tissue is replaced by scar tissue, leading to great loss of the contractile ability, which consequently may lead to heart failure. For these reasons, cardiac tissue regeneration may be the key to restoring the contractile ability of the heart after an injury caused by cardiovascular disease.^[^ ^100^ ^]^

3D electrospun structures can mimic the ECM of the myocardium, providing a nanofibrous microenvironment for cardiac cell adhesion, maturation, and function. Loosely packed 3D PPy scaffolds can enable stable electroactive cell−fiber construct formation and promote cell proliferation compared with a traditional 2D electrospun PPy fiber mesh.^[65a]^ Wet‐electrospun 3D alginate/gelatin hydrogel scaffolds were found to support the maturation of human iPS cell‐derived ventricular cardiomyocytes.^[42c]^ Likewise, 3D PCL nanofibrous scaffolds have been reported to directly promote cardiomyocyte differentiation through Wnt/β‐catenin signaling.^[^ ^101^ ^]^ 3D electrospun fibers have been manufactured from different ECM proteins, to provide suitable substrates for cardiac tissue regeneration. A scaffold containing the abundant ECM protein collagen maintained cardiomyocyte contractile function for a period of 17 days with high levels of desmin expression.^[^ ^102^ ^]^ ECM decellularized from the porcine ventricular tissue has been reported to provide natural cardiac ECM compositionally.[^9^, ^103^] Besides ECM proteins, the use of other naturally derived proteins, such as keratin^[^ ^104^ ^]^ and SF^[^ ^105^ ^]^ that are easy to obtain, have likewise been investigated. Fibrous scaffolds manufactured from SF have been reported to enhance cell commitment of adult rat cardiac progenitor cells.^[^ ^105^ ^]^

**Figure 7:**
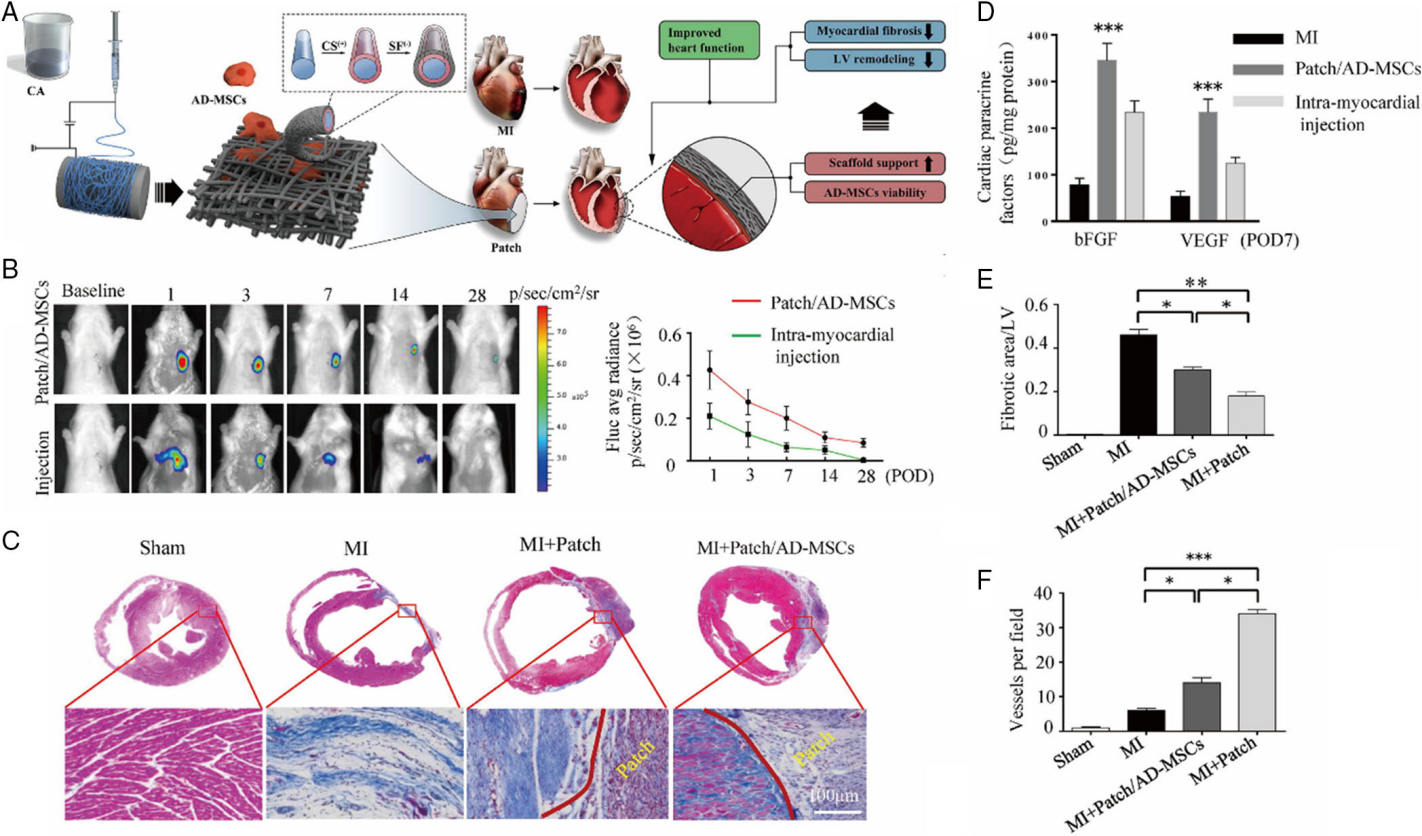
A) Schematic illustration of the cardiac patch fabricated by electrospinning to improve heart function. B) Viability of engrafted AD‐MSCs was tracked using bioluminescence imaging via built‐in Fluc 1, 3, 7, 14, 21, and 28 days postoperation. C) Chronic infarct size was determined using Masson's trichrome stain to label collagen scar tissue (blue) and cardiac muscle (red) 4 weeks postoperation. D) Cardiac paracrine factors were determined 24 h postoperation. E) The collagen area was calculated as the percentage of the total left ventricular myocardial area. F) The density of neovascularization was expressed as the quantity of arterioles per mm^2^. A–F) Reproduced with permission.^[^ ^106^ ^]^ Copyright 2018, Elsevier Ltd on behalf of Acta Materialia Inc.

Collectively, 3D electrospun scaffolds have been shown to exhibit improved performance for cardiomyocyte culturing compared with regular flat culture systems. A 3D cardiac patch‐based system has emerged as a potential regenerative strategy, as shown in **Figure** **7**A. In this study, SF‐modified CA 3D nanofibrous patches were used to restrain pathologic ventricular remodeling post‐MI by attenuating myocardial fibrosis.^[^ ^106^ ^]^ The patch/adipose tissue‐derived(AD)‐MSC group showed increased viability of engrafted AD‐MSCs (Figure 7B,C), expression of cardiac paracrine factors (Figure 7D), reduced fibrotic area (Figure 7E), and improved density of neovascularization (Figure 7F) compared with the intramyocardially injected AD‐MSC group and untreated groups. These results indicated that the 3D electrospun patch could be a good carrier, which allows high retention and survival of the engrafted AD‐MSCs.

As the human heart is a pulsating tissue, the mechanical properties are very important to consider when developing a cardiac patch. For this reason, cell‐laden polycarbonate−urethane (PCU) scaffolds have received much attention, due to their optimal Young's moduli (0.75 MPa, PCU electrospun on polyester fabric template), comparable with that of a human heart. The scaffold allowed prolonged spontaneous synchronous contractility of rat cardiac myoblasts on the entire engineered construct for 10 days in vitro at a near‐physiological frequency of ≈120 bpm.^[^ ^21^ ^]^

As a cardiomyocyte is a muscle cell, ES and directional arrangement are crucial. Therefore, conductive scaffolds have gained great interest in cardiac tissue regeneration in recent years. In one study, cardiac cells were seeded on nanogold‐coated PCL−gelatin scaffolds, which lead to cellular assembling of elongated and aligned tissues.^[^ ^107^ ^]^ In another study, conductive PLLA/PANI nanofibrous sheets were found to promote cardiac differentiation measured in terms of maturation index and fusion index.^[^ ^108^ ^]^ Others have focused on designing electrospun scaffolds to guide cell growth in precise directions. Cardiac cells cultured on aligned electrospun nanofibers were found to exhibit elongation and orientation of the α‐actin filaments,^[^ ^109^ ^]^ while simultaneously displaying high expression of genes encoding a number of sarcomere proteins, calcium‐handling proteins, and ion channels.^[^ ^110^ ^]^

**Figure 8:**
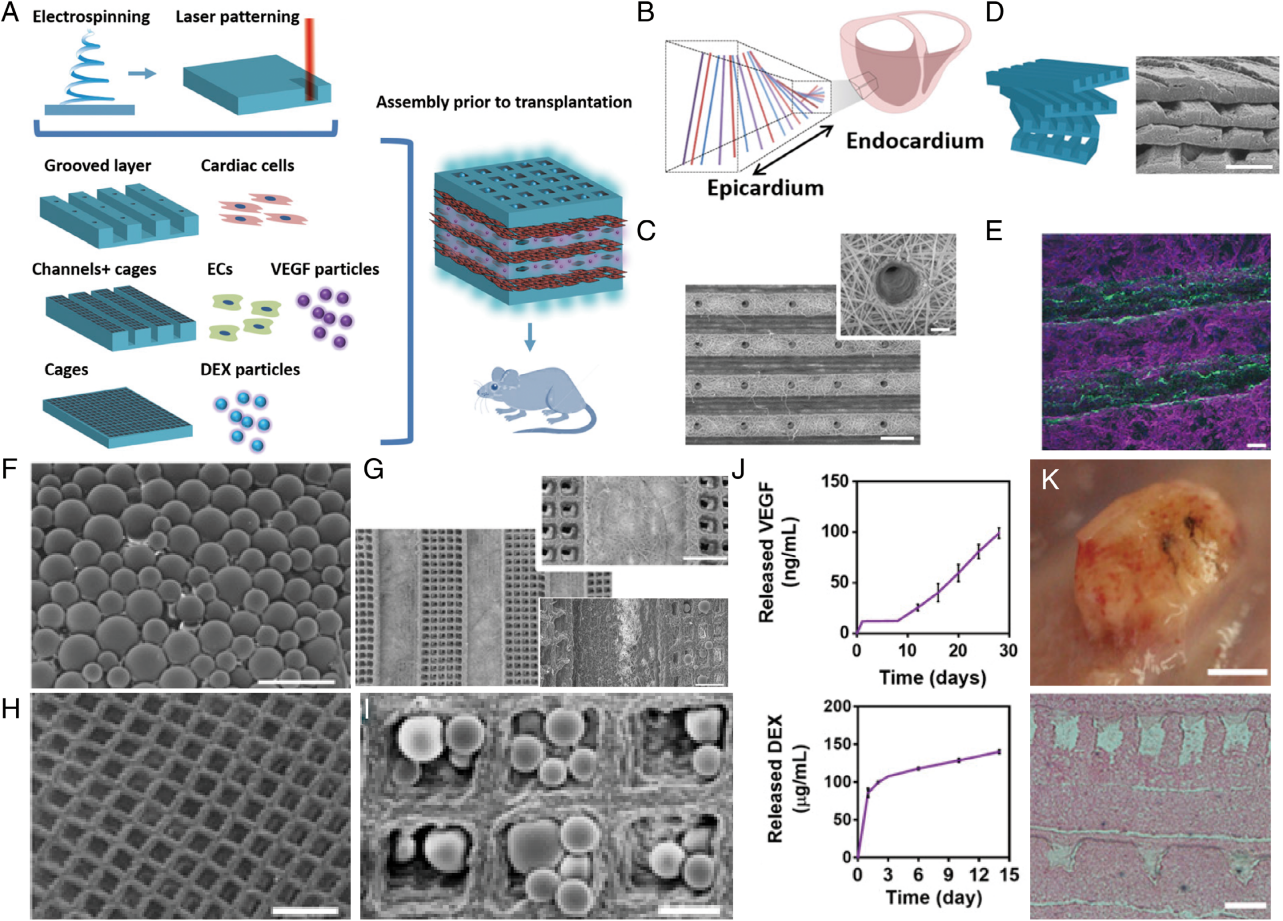
A) Steps in the bottom‐up approach to assembling a functional cardiac patch. B) Schematic illustration of a transmural block cut from the ventricular wall, showing macroscopic variation in fiber orientation across the wall. Grooved electrospun scaffolds with C) microholes and the D) micrographs of the grooved electrospun scaffolds stacked with a slight angle shift. E) Immunofluorescence images of α‐sarcomeric actinin (pink) and connexin 43 molecules (green) in cardiomyocytes cultured within grooved scaffolds. F) SEM micrograph of PLGA microparticles which acted as drug carriers. G) SEM micrographs of micropatterned tunnels and the cage‐like structures between them. SEM of micropatterned H) cage‐like structures and I) PLGA particles deposited on cage‐like structures. J) Cumulative release of VEGF and DEX. K) Vascularization of the patch with VEGF‐releasing particles 2 weeks after s.c. transplantation in rats and H&E staining of thin sections of the explanted patch. (Scale bars: 200 μm in C, and D; 20 μm in C Inset; 50 μm in E; 100 μm in F, H, G bottom, and G upper, K bottom; 500 μm in G; 50 μm in I; 5 mm in K upper). Reproduced with permission.^[1c]^ Copyright 2017, National Academy of Sciences.

Ultimately, multifunctional cardiac patches consisting of various tissue layers conducting different functions could be a promising solution for cardiac transplantation. A bottom‐up approach was proposed to assemble an impressively complex modular tissue (**Figure** **8**A).^[1c]^ Through analyzing collagen fiber orientation in adult rat hearts, Fleischer et al.^[1c]^ found that the alignment from the epicardial side to the endocardial side was a 100° shift (Figure 8B), which they used to guide the assembling of artificial multilayers by assembling several tissue layers on top of each other. Microholes (40 ± 0.8 μm) on the ridges of each layer were developed to ensure sufficient mass transfer (exchange of nutrients and oxygen) (Figure 8C). The grooved scaffolds were stacked with a slight angle shift to mimic the collagen fiber orientation in adult rat hearts (Figure 8D).

The cells in the grooved scaffolds were found to assemble into aligned cardiac cell bundles similar to that of the natural cardiac microenvironment with a higher expression of connexin 43 proteins (green color) due to the electrical coupling between adjacent cells (Figure 8E). Furthermore, double‐emulsion PLGA microparticles (Figure 8F) with VEGF were deposited into the cages of “channel + cages” layer (Figure 8G), thus creating a controlled release system for continuous supply of vascularization signals. Microtunnels with dimensions of 450 μm were patterned between cage‐like structures for endothelial cells to form large, closed lumens. In addition, Fleischer et al. designed another layer with cage‐like structures, and PLGA microparticles containing dexamethasone (DEX), an anti‐inflammatory agent, were scattered on top (Figure 8H,I) to attenuate the activation of macrophages and thereby decrease the immune response after transplantation. The PLGA microparticles were found to enable the long‐term release of VEGF and DEX (Figure 8J). A macroscopic view of the patches in rats and a cross sectioning of the patches revealed that the layered structure was maintained 2 weeks post‐transplantation (Figure 8K). The layers of the developed scaffold were found to mimic both the stiffness and the mechanical anisotropy of the heart muscle, hence improving the potential for the scaffolds to be integrated properly to the heart muscle.

**Table** **3** shows the characteristics of 3D electrospun scaffolds designed for cardiac tissue regeneration. Scaffold functionality in terms of bioactivity, aligned topography, and conductivity should still be improved to advance the potential of 3D electrospun scaffolds in the field of cardiac tissue regeneration. Based on the complexity of cardiac tissue, the ultimate goal of cardiac tissue regeneration is to achieve one scaffold with great diversity of functional elements to mimic the natural cardiac tissue.

**Table 3:** 3D electrospun scaffolds for cardiac tissue regeneration

Functional elements	Polymer substrate	Scaffolds structures	Fabrication techniques	Functional outcomes	Cell type	Refs.
Bioactivity	PCL/gelatin	Random nanofibers	Increasing spinning time	Cells differentiated into troponin T and myosin light‐chain 2a positive cardiomyocytes.	Murine‐induced pluripotent stem cells	[101]
	PEO (removable)/porcine cardiac ECM	Random submicrometer fibers	Increasing spinning time	Supported cell viability function and synchronized electrical activity in vitro. Biocompatible in vivo.	hMSC and human‐induced pluripotent stem cells	[9d]
	Alginate/gelatin hydrogel	Random nanofibers	Wet electrospinning	Improved cell adhesion, migration, proliferation, and maturation.	Human iPSC‐derived ventricular cardiomyocytes (hiPS‐CM)	[42c]
	Type 1 collagen	Random nanofibers	Increasing spinning time	Prolonged maintenance of cardiomyocyte contractile function on the scaffold.	Primary neonatal rat ventricular cardiomyocytes	[102]
	Cellulose/CS/SF	Random nanofibers	Increasing spinning time	Improvement of cardiac function and limitation of heart failure progression, deleterious ventricular remodeling restrained by nanopatch in vivo.	AD‐MSCs from mice	[106]
Bioactivity and aligned topography	Collagen/azacytidine	Aligned nanofibers	Increasing spinning time	Improved alignment, differentiation, and maturation into cardiomyogenic lineage cells.	Human bone marrow‐derived MSCs	[177]
Conductivity	PLLA/PANI/camphorsulfonic acid	Random nanofibers	Increasing spinning time; folding/rolling up	Increased numbers of myotubes number, a higher cell spreading, and alignment and cell−cell interactions.	H9C2 rat cardiomyoblasts	[108]
	PLLA (removable)/PPy	Fluffy random microfibers	Template‐assisted electrospinning	Increased cell proliferation.	Primary rat cardiomyocytes	[65a]
Conductivity and bioactivity	PCL−gelatin/AuNPs	Random submicrometer fibers	Increasing spinning time	Guided elongation of cardiac cells, higher contraction amplitudes and rates.	Neonatal rat ventricle myocytes	[107]
Pattern topography and mechanical property	PLGA/PCU	Patterned submicrometer fibers	Template‐assisted electrospinning	Supported cell adhesion and proliferation; guided anisotropic organization of cardiac‐like tissue; prolonged spontaneous synchronous contractility.	H9C2 rat cardiac myoblasts cell line	[21]
Aligned topography, mechanical property, and bioactivity	Albumin/VEGF/PLGA microparticles with anti‐inflammatory drugs	Microgrooves, Microchannels, and microcages	Increasing spinning time; laser patterning; layer‐by‐layer method	Mimicked both the stiffness and mechanical anisotropy of the heart muscle	Rat cardiac cells	[1c]

### 3.3 Bone Tissue Regeneration

As osteoporosis can increase the risk of bone fracture, it is the leading cause of broken bones among the elderly. For large bone defects beyond the ability of self‐regeneration, artificial scaffolds are necessary to bridge the gap, assist cell adhesion, and accelerate repair. This method is not only applicable for the elderly but may also be helpful to younger people by greatly decreasing the healing time. Importantly, advances in 3D electrospun scaffolds as bone substitutes are enhancing our ability to create ideal ECM mimicking structures for bone regeneration.^[^ ^111^ ^]^

The 3D electrospinning technology is a well‐established nano‐/microstrategy used to manufacture biomimetic fibrous constructs for bone tissue regeneration. A special 3D honeycomb‐like architecture has been proposed to support osteogenic differentiation by enhancing alkaline phosphatase (ALP) production, calcium deposition, and specific gene expressions.^[65b]^ In another study, conducted by Sankar et al., a 3D‐patterned electrospun scaffold was seeded with human AD stem cell spheroids. Osteodifferentiation was reportedly enhanced without the need for an osteoinductive culture medium.^[^ ^112^ ^]^ The scaffold shape can also be designed to match specific bones, such as the lumbar vertebra, as described by Su and coworkers.^[^ ^54^ ^]^

**Figure 9:**
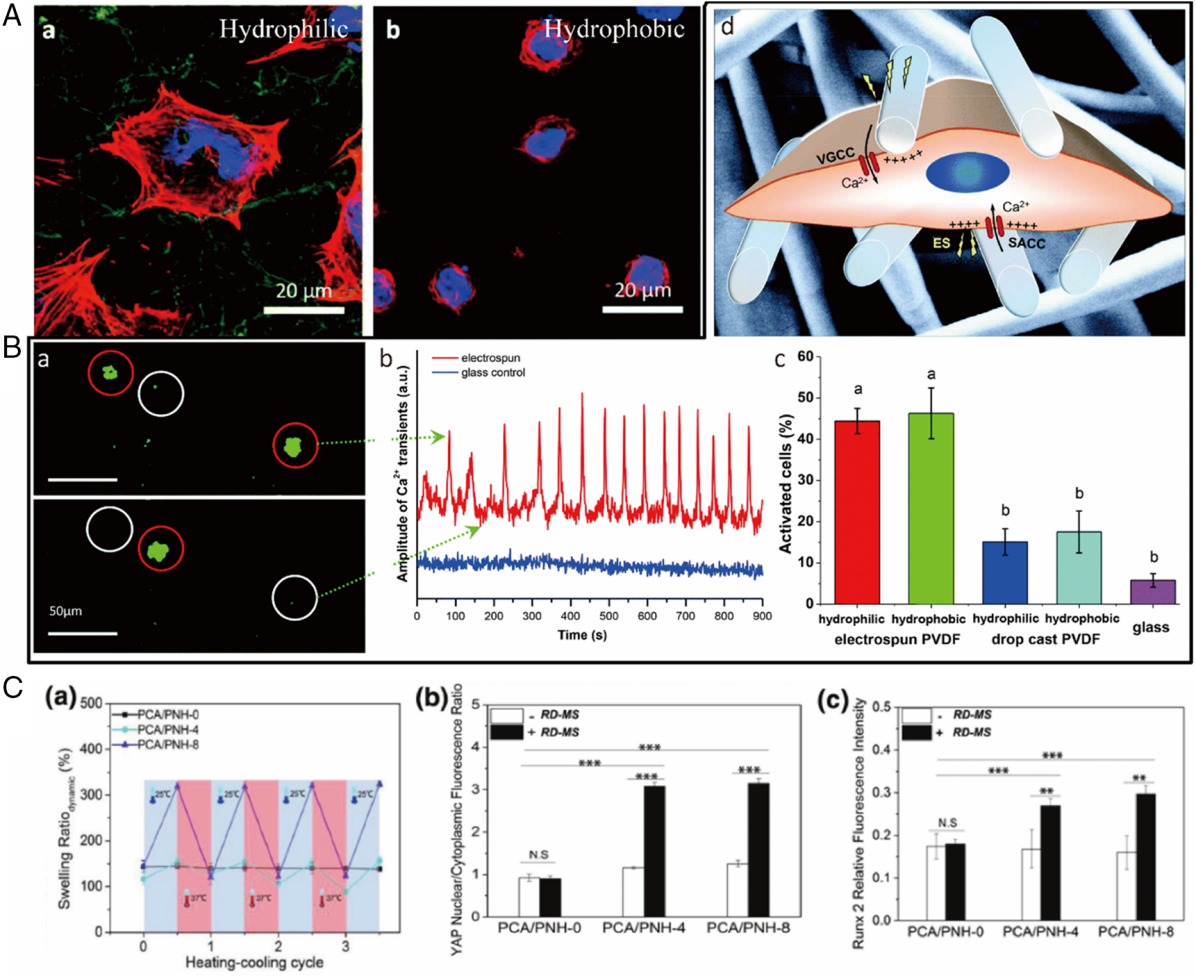
A) Saos‐2 cells cultured on (a) hydrophilic and (b) hydrophobic electrospun PVDF scaffolds for 3 days. B) PVDF piezoelectricity due to electrospinning‐induced intracellular calcium elevation in Saos‐2 cells. a) Snapshots of images showing changes in Ca^2+^ fluorescence intensity at different time frames. b) Ca^2+^ influx pattern of characteristic cells grown on hydrophilic electrospun scaffolds (red line) and the control glass coverslip (blue line). c) Percentage of activated cells grown on different PVDF scaffolds. d) Schematic representation of a cell cultured on hydrophilic electrospun PVDF scaffold; ES, coming from the 3D fibrous scaffold bending due to the cell forces applied upon attachment, causes the opening of plasma membrane channels (VGCC, SACC), which lead to an increase in calcium ions in the cytoplasm. A,B) Reproduced with permission.^[^ ^113^ ^]^ Copyright 2019, Royal Society of Chemistry. C) Temperature‐dependent changes a) in swelling ratio of crosslinked microfibrous hydrogels in response to temperature alternations. b,c) Ability of RD‐MS to alter hMSCs underlying YAP and RUNX2 signaling activation after 7 days. Reproduced with permission.^[^ ^115^ ^]^ Copyright 2018, Wiley‐VCH.

In vitro, 3D electrospun scaffolds support cell development. Human osteosarcoma cells cultured on piezoelectric hydrophilic electrospun PVDF scaffolds showed well‐defined actin stress fibers crossing the cell (**Figure** **9**A), and the scaffold was found to generate a local electric field that activated the osteoblasts (Figure 9B).^[^ ^113^ ^]^ The surface potential of PVDF fibers, furthermore, was found to regulate the production of mineralized collagen, a key component of the bone matrix, thus promoting the processes of bone regeneration.^[^ ^114^ ^]^ More complex electrospun scaffolds have allowed reversible dynamic mechanical stimulation for the study of cell response to mechanical forces. A thermocontrollable and stiffness‐tunable 3D electrospun microfibrous poly(*N*‐isopropylacrylamide) scaffold was used to submit cells to alternating cycles of mechanical stimulations by switching scaffold resistance between soft and stiff. The scaffold was reported to initiate cytoskeletal organization and cell shape deformation to activate the Yes‐associated protein (YAP) as well as the preosteogenic runt‐related transcription factor 2 (RUNX2), which is known to mediate mechanotransduction and differentiation (Figure 9C).^[^ ^115^ ^]^ Besides providing mechanical stimulation, reverse thermosensitive polymer fibers have also been used to support MSCs within 3D mechanically stable PCL.^[^ ^116^ ^]^

**Figure 10:**
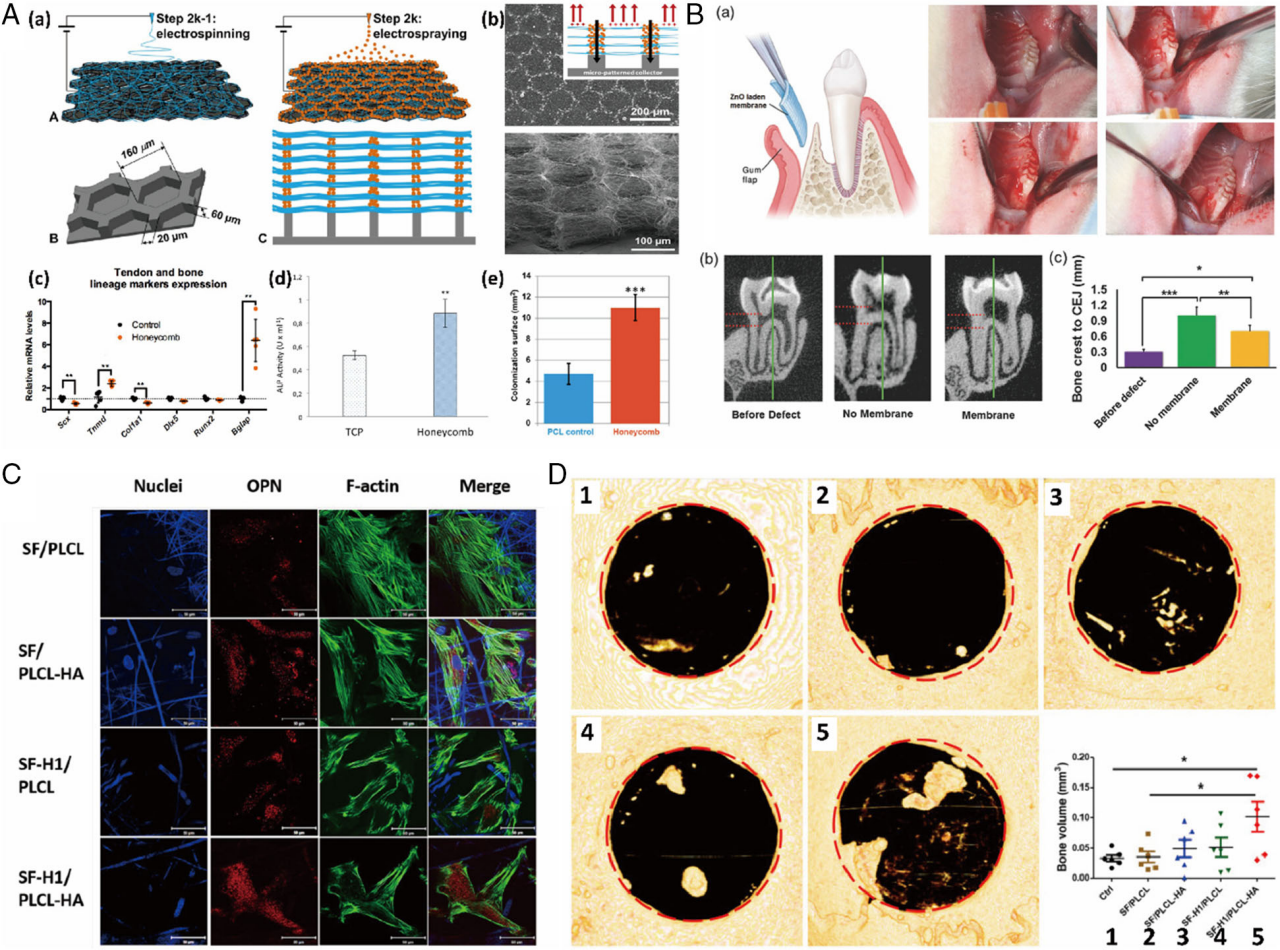
A) a) Design of honeycomb‐like scaffolds. b) Top view and side view of PCL‐HAp 3D electrospun scaffolds. c) Gene expression of tendon‐ and bone‐related markers in C3H10T1/2 cells cultured on the honeycomb‐like scaffolds compared with cells cultured without scaffold. d) ALP activity. e) Migration surface calculated by correlating cell surface in pixels to mm^2^. Reproduced with permission.^[^ ^33^ ^]^ Copyright 2018, American Chemical Society. B) Rat periodontal defect model. Reproduced with permission.^[^ ^121^ ^]^ Copyright 2017, Wiley‐VCH. C) Immunofluorescence images of osteogenic differentiation for hiPS‐MSCs on different core−shell scaffolds in osteogenic medium at week 3 (blue: 4′,6‐diamidino‐2‐phenylindole, DAPI; red: osteopontin, OPN; green: F‐actin). D) X‐ray micro‐computed tomography (μCT) analysis of the calvarial bone retrieved 8 weeks after implantation using various core−shell scaffolds in critical‐sized bone defects. C,D) Reproduced with permission.^[^ ^126^ ^]^ Copyright 2019, Elsevier.

Incorporating bioactive inorganic materials with electrospun polymers, mimicking the native bone ECM, has gained tremendous interest for bone tissue reconstruction throughout the years. Various modified and unmodified apatite minerals have been proven to resemble the physical properties of the natural bone tissue and promote osteogenic cell function. Beta‐tricalcium phosphate (β‐TCP),^[^ ^117^ ^]^ silicate‐containing hydroxyapatite,^[^ ^118^ ^]^ and boron doped hydroxyapatite (B‐HAp)^[^ ^119^ ^]^ have been proposed as bone‐like biomaterials mimicking the hierarchical architecture and chemical composition of the bone ECM. In one study, hydroxyapatite was incorporated in layer‐by‐layer electrospun honeycomb PCL fibers using electrospraying (**Figure** **10**Aa,b), mimicking the natural environment for maxillofacial bone reconstruction.^[^ ^33^ ^]^ Both reverse transcription‐quantitative real‐time PCR (Figure 10Ac) and ALP activity (Figure 10Ad) results clearly supported the positive effect of the honeycomb scaffolds on differentiation toward the bone lineage in the absence of any small‐molecule osteoinductive agents. Furthermore, the honeycomb structure resulted in significantly higher migration of cells from explanted bone tissue onto the scaffold compared with random PCL fibers. (Figure 10Ae). A natural occurring material, diatom shell, was incorporated into a 3D electrospun scaffold and was found to improve bone tissue regeneration.^[^ ^120^ ^]^ Another material that has been studied in regard to bone tissue regeneration is ZnO nanoparticles. Due to their osteoconductive and antibacterial properties, they were electrospun into PCL and demonstrated great potential for treatment of periodontal defects (Figure 10B).^[^ ^121^ ^]^

Another advantage of electrospun fibers is their capability to act as a great drug carrier or release system. In several studies, 3D electrospun scaffolds have been coated with bone morphogenetic protein‐2 (BMP‐2) on the surface by hydrogel or polydopamine (pDA)‐assisted immobilization and physical adsorption. This approach was biocompatible and osteoinductive in vitro and in vivo in a pilot study in rabbits.^[^ ^122^ ^]^ A low dose of recombinant human BMP‐2 has likewise been incorporated into 3D PCL/PLA scaffolds and promoted osteogenic differentiation and facilitated new bone formation after 6 weeks of implantation in vivo.^[^ ^123^ ^]^ In another study, rifampicin was incorporated in 3D electrospun scaffolds to successfully prevent bone infection, resulting from implant or orthopedic surgery.^[^ ^124^ ^]^ Alendronate, a nitrogenous bisphosphonate widely used in the therapy of metabolic bone diseases, was incorporated into electrospun scaffolds and promoted osteogenesis‐related gene expression in human fetal osteoblasts.^[^ ^125^ ^]^ Finally, Xu et al. produced electrospun SF/poly(L‐lactide*‐co‐*ε‐caprolactone) (PLCL) core−shell fibers that were used to dual‐deliver the osteoinductive peptide H1 from the core and HAp from the shell for collectively enhancing osteogenesis both in vitro and in vivo. This was proven by increased expression of osteopontin (Figure 10C) and induced bone mineralization (Figure 10D) compared with control scaffolds.^[^ ^126^ ^]^

**Table** **4** shows different 3D electrospun scaffolds used for bone tissue regeneration. In the field, the cues of bioinorganic, electromechanical, and mechanical stimulation have been the main research direction in the recent years. Scaffolds with drug‐carrying or releasing properties have also gained tremendous attention with a large number of published reports. All the electrospun scaffolds implanted in animal models mentioned in this Review were uncellularized, designed to stimulate the animal's own cells to produce new bone. However, it would be ideal to stimulate cells in vitro to form artificial bone with the same properties as native bone. The shape of artificial bone can be controlled by the shape of electrospun scaffolds by combining it with either 3D printing, MEW, NFE, or field‐assist electrospinning.

**Table 4:** 3D electrospun scaffolds for bone tissue regeneration

Functional elements	Polymer substrate	Scaffolds structures	Fabrication techniques	Functional outcomes	Cell type	Refs.
Bioactivity	PCL/GelMA hydrogels	High‐porosity microfiber scaffolds	Electrospinning writing	Stiffness and elasticity resembled that of articular cartilage tissue, chondrocytes survived and were functional.	Human chondrocytes	[53b]
	PCL/poly(ethylene glycol)/heparin hydrogel	Fibrous network	Electrospinning writing	Biomechanical properties close to human cartilage, chondrocyte viability.	Primary human articular chondrocytes	[178]
Bioactivity and aligned topography	PCL/methacrylated gelatin	Aligned microfibers	Increasing spinning time; crosslinkage	Structure resembled native tendon tissue; promoted cell elongation	Human adipose‐derived stem cells	[179]
	PCL/carboxymethyl CS/sodium alginate	Random and aligned microfibers	Increasing spinning time	No significant cytotoxicity; facilitation of osteoblast adhesion; upregulation of the early expression of osteogenic genes ALP and Runx2.	Murine MC3T3‐E1 osteoblast cells	[180]
Bioinorganic materials	PCL/SiHAp microparticles	Random and aligned microfibers	Increasing spinning time	Improved cellular viability and bone in‐growth.	hMSC	[118]
	Poly(butylene adipate*‐co‐*terephthalate)/B‐HAp	Random submicrometer fibers	Wet electrospinning	Higher ALP activity and amounts of collagen and calcium; higher expression levels of both early‐ and late‐stage osteogenic genes.	Human bone marrow‐derived stem cells (hBMSCs)	[119]
	PCL/HAp	Honeycomb‐like structure	Template‐assisted electrospinning, layer‐by‐layer electrospinning	Guided the migration of differentiated bone cells.	Embryonic murine cell line C3H10T1/2	[33]
	PLLA/lactic acid (LA)/β‐TCP	Fluffy random nanofibers	Self‐assembly electrospinning	Cells penetrated the scaffold and showed ALP activity.	Human MSC‐derived preosteoblast cells	[117]
Bioinorganic materials and bioactivity	PLLA/pDA/gelatin/biominerals	Random nanofibers	Increasing spinning time	Enhanced cell attachment and expression of osteogenic genes.	Human adipose tissue‐derived stem cells	[181]
	PCL/Chondroitin sulfate and sol−gel‐derived bioactive glass	Random mircofibers	Increasing spinning time	Accelerated cell integration into subchondral bone and remineralization processes.	Goat chondrocytes	[182]
Bioinorganic, patterned topography	PLGA/collagen/nHAp	Random and patterned nanofibers	Template‐assisted electrospinning	Differentiation of MSC spheroids into osteogenic lineage even in the absence of osteoinduction medium.	Human MSCs from adipose tissue	[112]
Bioinorganic materials, conductive, aligned topography	PLA/multiwalled carbon nanotubes and nanohydroxyapatite	Random submicrometer fibers	Layer‐by‐layer electrospinning	Supported cell viability.	Primary human osteoblast cells	[183]
Electromechanical stimulation	PVDF with oxygen plasma treatment	Random nanofibers	Increasing spinning time	Electromechanical stimulation produced intracellular calcium transients without the need of an external power source.	Human osteosarcoma Saos‐2 cells	[113]
	PVDF applied voltage polarities	Random submicrometer fibers	Increasing spinning time	Controlled cell proliferation and collagen mineralization.	Human osteoblast‐like cell line MG63	[114]
Mechanical stimulation	Acryloyl carbonated polycaprolactone/a copolymer of *N*‐isopropylacrylamide and 2‐hydrox‐yethyl methacrylat	Random microfibers	Increasing spinning time	Initiated cytoskeletal organization and cell shape deformation, increased cell spreading and polarization.	hMSC	[115]
	PCL/poly(ethylene glycol)‐poly(*N*‐isopropylacrylamide)	Random microfibers	Increasing spinning time	Enhanced chondrogenic differentiation.	hMSC	[116]
Drug release	PCL/PLGA/rifampicin	Random nanofibers	Increasing spinning time	Supported cell growth; prevented bacterial infection.	Human osteoblasts	[124]
Drug release, bioactivity	PCL/CS/bone morphogenetic protein BMP‐7	Random nanofibers	Template‐assisted electrospinning	Accelerated bone mineralization and regeneration in vivo.	Human primary osteoblasts and human bone marrow MSCs	[184]
	PCL/PLLA/hyaluronic acid hydrogel/BMP‐2	High‐porosity microfiber scaffolds	Electrospinning writing	Upregulation of bone markers such as osteopontin, osteocalcin, and collagen 1A1.	Human primary osteoblast cells	[122b]
Drug release, mechanical properties	PCL/PLA/recombinant human BMP‐2	Random nanofibers	Increasing spinning time; postfreeze drying	Higher cell viability and osteogenic differentiation.	hMSCs	[123]
Drug release, bioinorganic materials	PCL/nano‐HAp/alendronate	Patterned nanofiber	Template‐assisted electrospinning; layer by layer	Promoted osteogenesis‐related gene expression.	Human fetal osteoblasts	[125]
	Poly(hydroxybutyrate*‐co‐*hydroxyvalerate)/PCL/cefuroxime axetil/PUL/diatom shells	Random microfibers	Wet electrospinning	Improved cell viability and cell spreading.	Human primary sarcoma cell line	[120]
Drug release, bioinorganic materials, bioactivity	Nano‐HAp/PLLA/gelatin/pDA/BMP‐2‐derived peptides	Random nanofibers	Increasing spinning time; postfreeze drying	Supported cell adhesion, proliferation, migration, and differentiation; promoted bone regeneration in a rat model of cranial bone defect.	BMSCs	[122a]
	PLCL/SF/HAp/osteogenic inductive peptide H1	Random nanofibers	Increasing spinning time	Promoted cell proliferation, osteoblastic differentiation, and bone tissue formation.	Human‐induced pluripotent stem cell‐derived MSCs (hiPS‐MSCs)	[126]

### 3.4 Vascular Tissue Regeneration

If tissue regeneration of tissues larger than 200 μm ever is to succeed, the field needs to overcome the challenge of creating fully functional blood vessels. Vascularization of tissues is essential for supplying the cells with nutrients and oxygen and to help remove waste products.^[^ ^127^ ^]^ Su and coworkers developed an artificial multihelix electrospun PCL/PCL + PEO scaffold.^[^ ^128^ ^]^ Here, the seeded human umbilical vein endothelial cells (HUVECs) were reported to exhibit a preferential cell distribution of around 86% on the PCL side rather than the PCL + PEO side, resulting in a distinctive 3D Janus cellular pattern with increased vinculin and phosphorylated‐focal adhesion kinase (pFAK) expression. Kim et al. developed a vascularized 3D tissue by stacking HUVEC cell sheets with skeletal myoblasts and fibroblast cell sheets in a layer‐by‐layer construction.^[59c]^ In another work, the thickness of a vascularized tissue construct was controlled by the thickness of the electrospun scaffold through the increasing layer number.^[^ ^129^ ^]^ Besides solution electrospinning, MEW has been used for coculture of HUVECs and normal human dermal ﬁbroblasts (NHDF) through cell accumulation technique, which allowed the formation of capillary‐like network structures.^[^ ^130^ ^]^

**Figure 11:**
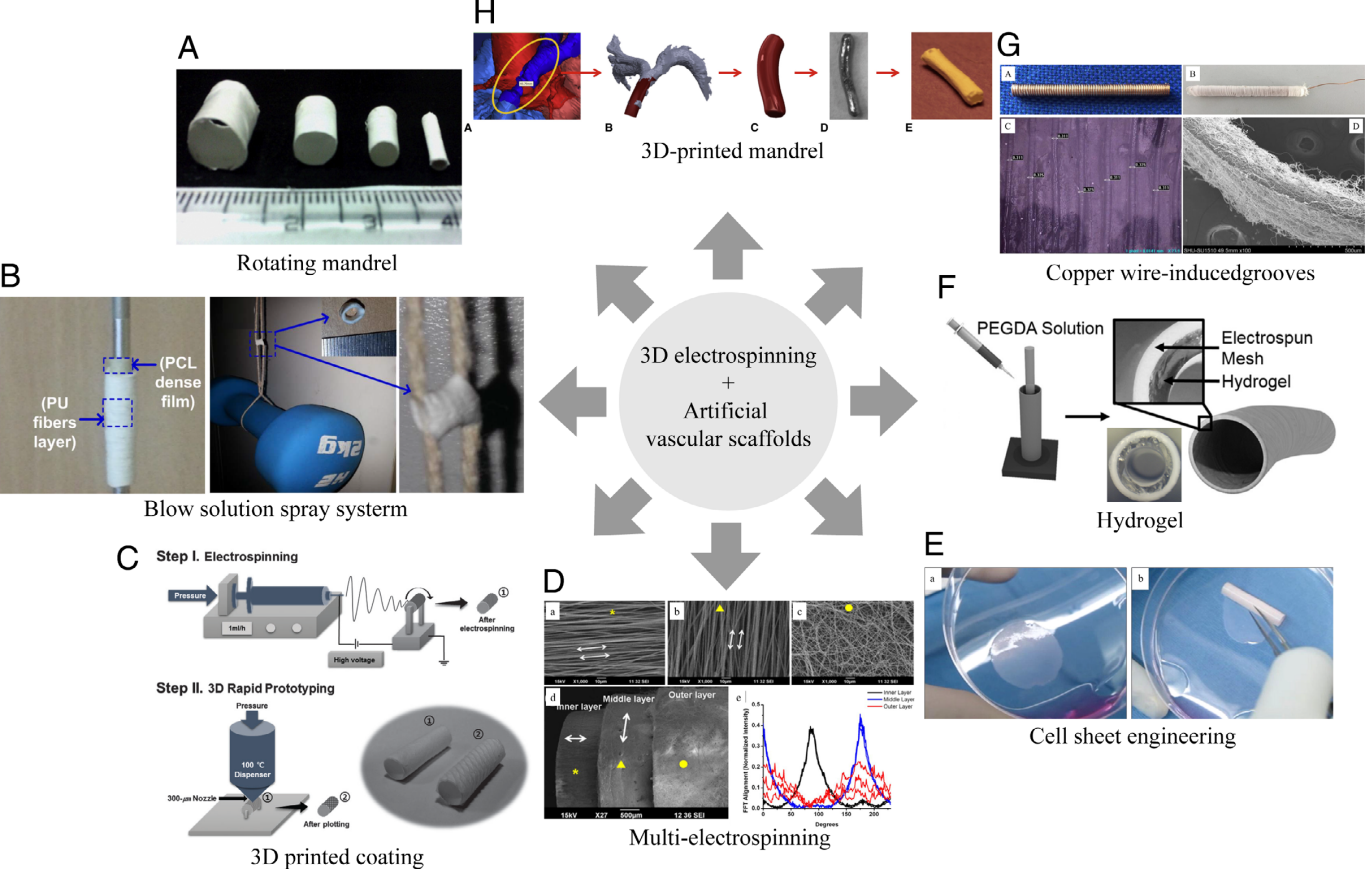
A) PLLA/gelatin electrospun tubes with an inner diameter range of 2–6 mm and inside axially aligned morphology fabricated by rolling method. Reproduced with permission.^[^ ^131^ ^]^ Copyright 2015, Elsevier. B) Digital image of fabricated biphasic tubular scaffold, with PCL forming an inner lamina and electrospun PU forming an outer lamina. Images showing a 2 kg dumbbell hanging on a PCL/PU tube scaffold. Reproduced with permission.^[64b]^ Copyright 2018, Elsevier. C) Schematic illustration of the process used to fabricate 3D tubular artificial vascular scaffolds combining electrospinning with 3D‐printed coating. Reproduced with permission.^[^ ^134^ ^]^ Copyright 2015, Royal Society of Chemistry. D) Longitudinal view of photographic and scanning electron microscopic images of electrospun trilayered tubular scaffolds as well as the fast FFT analysis. Reproduced with permission.^[^ ^135^ ^]^ Copyright 2018, Elsevier. E) Prefabricated cell sheets wrapped around an electrospun scaffold. Reproduced with permission.^[^ ^138^ ^]^ Copyright 2015, Elsevier. F) Schematic of multilayer graft fabricated by electrospun tubular scaffold and hydrogel. Reproduced with permission.^[^ ^140^ ^]^ Copyright 2018, Elsevier. G) The photo of an electrospinning collector with copper wire wound around it and the interior copper wire being easily pulled out. Grooves and cross‐sectional morphology of the scaffold. Reproduced with permission.^[63b]^ Copyright 2016, Elsevier. H) Schematic depicting the proposed process for manufacturing patient‐specific tissue‐engineered vascular grafts. Reproduced with permission.^[^ ^20^ ^]^ Copyright 2016, Elsevier.

Regarding artificial blood vessel architecture, 3D electrospinning is a simple and ideal method to fabricate tubular‐shaped biodegradable scaffolds. Such scaffolds have been fabricated based on either rotating mandrel collectors or post‐treatments. In one study, engineered vascular grafts with diameters matching vascular vessels were prepared for vascular reconstruction by simply adjusting the diameter of the mandrel (**Figure** **11**A).^[^ ^131^ ^]^ Another strategy applied for obtaining tubular constructs was an automated fabrication strategy, which rolled 2D matrices into 3D tubular constructs by continuously bonding different functional layers (cells, hydrogels, and scaffold biomaterials) with varying diameters.^[^ ^132^ ^]^

**Figure 12:**
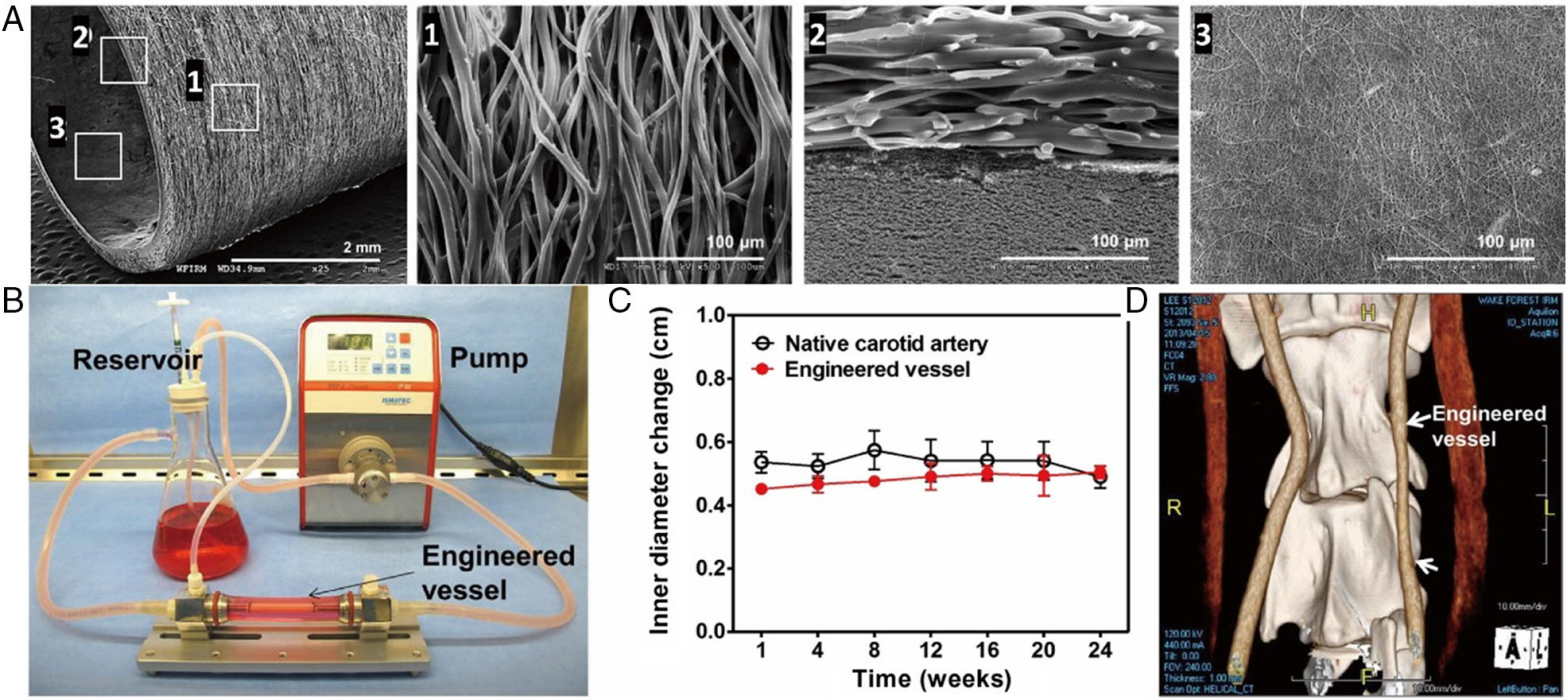
A) SEM images of entire layer, outer layer, cross‐sectional interface and inner layer of the bilayered vascular scaffold. B) Bioreactor setup consisting of a computer‐programmed gear pump, flow reservoir, and the bioreactor housing unit. C) Inner diameter measurement of the transplanted engineered blood vessels revealed a stable lumen caliber for the duration of the 6‐month follow‐up. D) A representative CT scan shows the absence of an aneurysm along the entire length of the transplanted engineered blood vessel (arrows). A–D) Reproduced with permission.^[^ ^137^ ^]^ Copyright 2017, Elsevier.

In addition to scaffold shape, mechanical properties are key factors to consider when developing material for vascularized tissue regeneration. In one study, PU was electrospun onto an airbrushed PCL tube to mechanically reinforce the scaffold. The tensile strength and Young's modulus of the reinforced scaffolds were reported to be 67.5 ± 2.4 and 1039 ± 81.8 MPa, respectively, allowing a 2 kg dumbbell to hang from the scaffold without breaking it (Figure 11B).^[64b]^ Materials like polyamide‐6 and GO have likewise been proven to reinforce the mechanical and physical properties of electrospun scaffolds.^[^ ^131^, ^133^ ^]^ By combining electrospun PCL with 3D‐printed PCL, both surface morphology and mechanical properties were found to be suitable for vascular reconstruction (Figure 11C).^[^ ^134^ ^]^ Another approach to obtain mechanically reinforced scaffolds is using multiple electrospun layers with different architectures. Such a scaffold was designed using longitudinally aligned nanofibers (inner and outer layer) and radially aligned nanofibers (middle layer) and successfully mimicked native artery structure (Figure 11D).^[^ ^135^ ^]^ Similarly, the NFE technique combined with electrospraying and electrospinning produced a mechanically reinforced three‐layered scaffold with highly aligned strong fibers, which provided appropriate mechanical support for HUVECs in vitro and allowed infiltration of host cells after implantation into the abdominal aorta in vivo.^[^ ^136^ ^]^ Ju and coworkers^[^ ^137^ ^]^ manufactured a bilayered electrospun and cellularized vascular scaffold and assessed its preclinical feasibility in sheep. The electrospun scaffold was composed of randomly orientated fibers in the inner layer and aligned fibers in the outer layer (**Figure** **12**A). The scaffold was seeded with smooth muscle cells (SMCs) and endothelial cells and preconditioned using a pulsatile bioreactor system (Figure 12B) prior to transplantation. The cellularized vascular constructs were found to maintain a high degree of graft patency with a constant luminal diameter (Figure 12C), structural integrity with compliance (Figure 12D), and contractile properties without eliciting an inflammatory response after a 6‐month implantation period. Consequently, this scaffold was reported as a clinically applicable alternative to traditional prosthetic vascular graft substitutes.

Ahn et al. improved the cell seeding efficiency on vascular‐like electrospun PCL and collagen type I fibers, by prefabricating an SMC sheet. The cell sheet was wrapped around the electrospun vascular scaffolds and found to significantly enhance not only the cell seeding efficiency, but also the maturation process and the cell‐to‐cell junctions compared with cells seeded directly on the electrospun fibers, yielding an implantable vascular graft (Figure 11E).^[^ ^138^ ^]^ Wang et al.^[^ ^139^ ^]^ used the molecule‐releasing properties of electrospun fibers. Here, resveratrol, a natural polyphenol, was incorporated into electrospun PCL fibers, allowing sustained and controlled release that was found to enhance the vascular regeneration process by promoting migration of endothelial cells and tube formation. Another study conducted by Post et al.^[^ ^140^ ^]^ showed that designing a layered cell‐free hydrogel system likewise led to rapid endothelialization (Figure 11F). Hydrogels have also been used to coat electrospun scaffolds with copper wire‐induced grooves (Figure 11G) using a so‐called dip‐coating method. This technique promoted the attachment of HUVECs.^[63b]^ The hydrogels can increase hydrophilicity, biocompatibility, and mechanical strength and therefore improve affinity between cells and the electrospun nanofibers.

To overcome the challenge of anatomic differences between individual patients, Fukunishi et al. developed a patient‐specific vascular graft. A 3D‐printed patient‐specific stainless steel graft was used as an electrospinning collector, allowing both SMC engraftment and endothelialization; hence, this method was found to be a promising technology for future vascular tissue regeneration. (Figure 11H).^[^ ^20^ ^]^

**Table** **5** shows the 3D electrospun scaffolds for vascularized tissue regeneration highlighted in this Review. There are relatively few studies published on biological activity, cellular interaction, and drug release compared with other fields of tissue regeneration. This might, however, be explained by the intense focus on optimizing scaffold shape and mechanical properties for vascularized tissue regeneration. For this field to reach its full potential, future studies should closely examine both vascular cell response and scaffold architecture.

**Table 5:** 3D electrospun scaffolds for vascular tissue regeneration

Flat or tubular	Tubular strategy	Functional elements	Polymer substrate	Scaffolds structures	Fabrication techniques	Functional outcomes	Cell type	Refs.
Flat scaffolds	–	3D architecture	Poly(carbonate urethane)	Random submicrometer fibers	Increasing spinning time	Promoted cell differentiation toward the synthetic vascular SMC phenotype.	Human coronary artery SMC	[185]
			PCL/PEO	Janus microfibers	Wet electrospinning	Higher focal adhesion, enhanced cell proliferation, and elongation.	HUVECs	[128]
			PCL	Highly structured microfiber scaffolds	Electrospinning writing	Formation of capillary‐like structures; increased VEGF secretion.	HUVECs and NHDF	[130]
		Bioactivity	PLGA/collagen	Random microfibers	Increasing spinning time; embedded with collagen hydrogel	Formation of a prevascularised construct, the artificial capillaries anastomosed with host vasculature of grafted mice.	HUVECs	[186]
			PVA/gelatin	Random nanofibers	Increasing spinning time	Promoted cell adhesion and migration.	Rat SMCs and HUVECs	[187]
Tubular scaffolds	Rolling collector	3D architecture	PCL/PU	Random submicrometer fibers	Template‐assisted electrospinning	High cell viability.	Human endothelial cell line (EA.hy926)	[64b]
			PCL/polyamide‐6	Random nanofiber	Template‐assisted electrospinning	Supported endothelial cell migration and cellular infiltration.	EA.hy926 EC	[133a]
			Polyglycolic acid and PLCL	Random nanofiber	Template‐assisted electrospinning	Addressed the diverse anatomic requirements of individual patients.	Cell free	[20]
		Conductivty	Thermoplastic polyurethane (TPU)/GO	Random nanofiber	Template‐assisted electrospinning	Enhanced HUVEC viability and attachment.	Mouse fibroblast and HUVECs	[155b]
		Bioactivity	PLLA/gelatin	Random nanofiber	Template‐assisted electrospinning	Improved mechanical properties.	Cell free	[133c]
			PCL/PVA and sodium alginate	Random nanofiber membrane with grooves structure	Template‐assisted electrospinning	Mechanical and structural properties resembled native vessels and promoted cell integration, adhesion, and growth.	HUVECs	[63b]
		Drug release	PCL/resveratrol	Random microfiber	Template‐assisted electrospinning	Increased cell migration, promoted endothelialization and vascular regeneration.	HUVECs	[139]
		Patterned topography	PCL	Random nanofiber	Template‐assisted electrospinning; layer‐by‐layer electrospinning	Improved surface morphology and mechanical properties.	Cell free	[134]
		Aligned topography	PCL/polyethylene glycol	Highly aligned microfibers, longitudinally oriented nanofibers, and random microfibers	Electrospinning writing; layer‐by‐layer electrospinning; template‐assisted electrospinning	Enhanced cell proliferation and migration; adequate porosity and cell penetration.	HUVECs	[136]
		Aligned topography, bioactivity	PCL/type I collagen	Aligned microfiber	Template‐assisted electrospinning	High cell seeding efficiency, supported a mature smooth muscle layer	SMCs from sheep femoral artery	[138]
			PCL/collagen	Aligned microfibers (outer layer) and random nanofibers (inner layer)	Template‐assisted electrospinning; layer‐by‐layer electrospinning	Sustained structural integrity with a high degree of graft patency without eliciting an inflammatory response in an ovine model of carotid artery injury.	Sheep endothelial progenitor cell‐derived endothelial cells and SMCs	[137]
	Postrolling method	Aligned topography, bioactivity	PLLA/gelatin	Aligned and random nanofibers	Increasing spinning time	Increased viability and proliferation; elongation of cells along the scaffold.	HUVECs and SMCs	[131]
		Aligned topography, drug release	Poly(hydroxy butyrate*‐co‐*hydroxy valerate) and PVA/VEGF/platelet factor concentrate	Longitudinally aligned nanofibers (inner layer), radially aligned nanofibers (middle layer), longitudinally aligned and random multiscale fibers (peripheral layer)	Layer‐by‐layer electrospinning	Cell elongation and alignment along the direction of fibers; expressed endothelial markers	HUVECs, SMCs, and MSCs	[135]
	Automated fabrication system	Bioactivity	Collagen/PNIPAAm/polyethylene terephthalate	Random nanofibers	Increasing spinning time; rolling up	Formation of structures resembling mammary artery.	NIH3T3 mouse embryonic fibroblasts, HUVECs, BJ6 fibroblast cells and human colon adenocarcinoma (CACO‐2) cells; SMCs	[132]
	Shape‐memory	3D architecture	PCL/GelMA/poly(lactide*‐co‐*trimethylene carbonate)	Random nanofibers	Increasing spinning time	Supported endothelial cell attachment and viability.	HUVECs	[188]

### 3.5 Skin Tissue Regeneration

**Figure 13:**
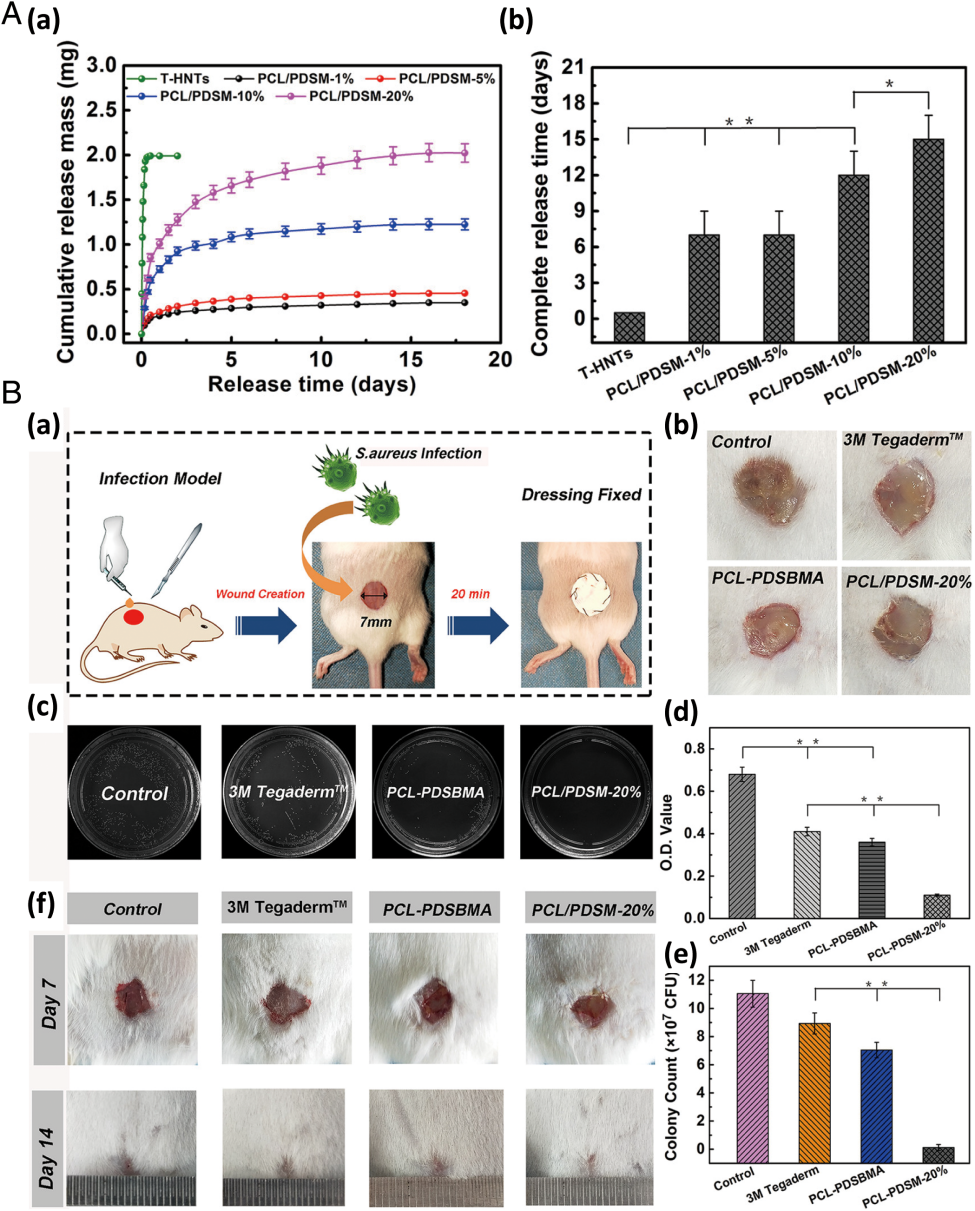
A) Cumulative drug release profile (a) and complete drug release time (b) of the T‐HNTs and composite membranes. B) In vivo antibacterial investigations of the T‐HNT‐doped composite membranes (PCL/PDSM‐20%). a) illustration of the bacterial infection animal model; b) images of *S. aureus*‐infected wounds covered with different dressings after 3 days of incubation; c) bacterial incubation results of different wound exudates; and d) optical density (O.D). value and e) colony count of different wound exudates after 12 h of incubation. f) Wound healing evaluation of the bacterial models. *P < 0.05, **P < 0.01. A,B) Reproduced with permission.^[^ ^149^ ^]^ Copyright 2019, American Chemical Society.

3D electrospun scaffolds with high porosity and surface‐to‐volume ratio have been shown to exhibit tremendous potential for wound healing and injured skin tissue regeneration. One application of electrospun materials is to create antibacterial wound dressings. As bacterial infections may result in increased exudate at the wound site, a wound dressing carrying antibacterial drugs is a direct method to overcome this challenge. In one study, poly(3‐hydroxybutyric acid) and gelatin loaded with curcumin were electrospun onto a keratin−fibrin−gelatin hydrogel substrate. This dual system was found to facilitate tissue regeneration, while preventing infection at the transplant site.^[^ ^141^ ^]^ Similarly, thymoquinone and antibiotic tetracycline hydrochloride^[^ ^142^ ^]^ were incorporated into 3D electrospun scaffolds and prevented common clinical infections and promoted healing.^[^ ^143^ ^]^ Several other antibacterial materials, such as the peptide OH‐CATH30 (OH‐30),^[^ ^144^ ^]^ CS,^[^ ^145^ ^]^ medicinal plant extracts,^[^ ^146^ ^]^ and silver nanoparticles,^[^ ^147^ ^]^ have likewise been investigated for this purpose, with promising results. As zwitterionic polymers have excellent antibiofouling abilities, they have also been used for wound‐dressing purposes.^[^ ^148^ ^]^ As an example, electrospun PCL membranes were functionalized with a zwitterionic polymer. To this system, halloysite nanotubes were like sustained drug carriers (**Figure** **13**A) of the broad‐spectrum antibiotic tetracycline hydrochloride. The scaffolds were found to possess great selective biocompatibility, promoting platelet and mouse fibroblasts L929 cell adhesion and decreasing accumulation of both plasma proteins and bacteria. The scaffold increased skin regeneration compared with the commercial 3M Tegaderm^TM^ film (Figure 13B).^[^ ^149^ ^]^ He et al.^[^ ^150^ ^]^ developed a scaffold composed of quaternized chitosan graft‐polyaniline (QCSP) in PCL and found it to significantly accelerate the wound‐healing process in mice compared with the commercial Tegaderm^TM^ film. Finally, the analgesic drug, lidocaine hydrochloride, was incorporated in an electrospun scaffold together with the anti‐inflammatory agent curcumin. The developed scaffold showed great antibacterial performance in vitro and has great potential in wound care applications, though it has not yet been tested in vivo.^[^ ^151^ ^]^

**Figure 14:**
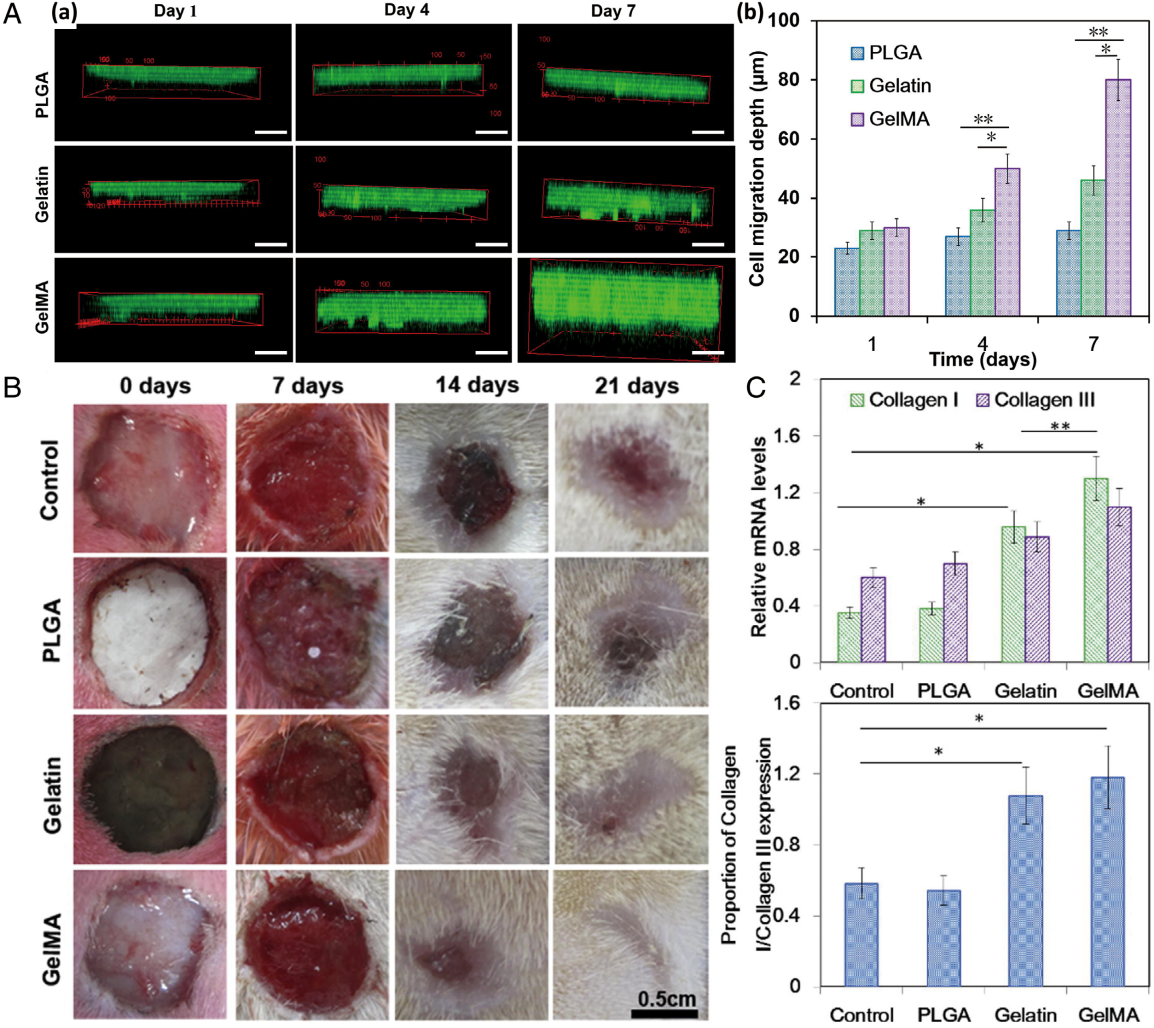
A) Cell migration into the electrospun scaffolds. B) In vivo wound healing using PLGA, gelatin, and GelMA‐10 scaffolds. Images of wound beds healed using different electrospun fibrous scaffolds. C) Relative mRNA levels of collagen I and collagen III after 21 days of operation using different scaffolds. A–C) Reproduced with permission.^[155a]^ Copyright 2016, Elsevier.

Other types of wound dressings focus on accelerating cell proliferation, rather than preventing infection, to induce repair of the damaged skin. Without any anti‐inflammatory drugs, electrospun PCL reportedly provides a suitable 3D environment for differentiation of melanocytes and can be a promising candidate for treatment of skin disorders.^[^ ^152^ ^]^ Combining PCL with other elements could further enhance skin repair. For instance, heparin and fibroblast growth factor‐2‐modified PCL have been found to promote the formation of distinct fibroblast and keratinocyte layers.^[^ ^153^ ^]^ Collagen‐coated PCL scaffolds have likewise been found to increase attachment and proliferation rates of human endometrial stem cells compared with pure PCL.^[^ ^154^ ^]^ Other types of 3D electrospun scaffolds composed of either GelMA^[^ ^155^ ^]^ or CA/PUL^[^ ^156^ ^]^ have shown promising results in skin tissue regeneration applications. Compared with PLCL and gelatin scaffolds, the GelMA scaffolds were found to promote cell proliferation (**Figure** **14**A), reduce remaining wound area (Figure 14B), and increase expression of the ECM protein collagen I (Figure 14C).^[155a]^

SF is another biomaterial that has been widely investigated for its promising properties for skin tissue regeneration. Electrospun SF scaffolds have been fabricated using either cold‐plate electrospinning or by dropping NaCl crystals, which have resulted in scaffolds with high porosity and controllable thickness, indicating that these scaffold types are potential candidates for artificial skin reconstruction.^[^ ^29^, ^157^ ^]^ SF has, furthermore, been used in combination with other polymers or nanoparticles. Incorporation of gold nanoparticles (AuNPs) into tissue scaffolds, has been shown to enhance mechanical stability. SF/PEO/AuNP 3D matrices is one such example and was successfully tested in a rat model of full‐thickness skin wound.^[^ ^158^ ^]^ In another study, 3D PCL/SF scaffolds have been found to increase the wound healing rate and collagen deposition in rats compared with PCL alone.^[28b]^ Finally, SF combined with decellularized human amniotic membrane (AM) has been found to be an efficient antiscarring wound‐dressing material for third‐degree burn wounds.^[^ ^159^ ^]^

**Table** **6** shows some of the 3D electrospun scaffolds developed for skin tissue regeneration. The main goals are to achieve both bacteriostasis and cell proliferation and migration by functionalizing the wound‐healing scaffolds with antibacterial components, drug‐releasing carriers, and bioactive materials. The electrospun mat was also designed with human skin patterns to mimic the actual human skin.^[^ ^160^ ^]^ The development of portable electrospinning devices with a hand‐held spinneret could allow the scaffold to be directly applied to the damaged skin, therefore simplifying the fabrication process.^[^ ^161^ ^]^

**Table 6:** 3D electrospun scaffolds for skin tissue regeneration

Functional elements	Polymer substrate	Scaffolds structures	Fabrication techniques	Functional outcomes	Cell type	Refs.
3D architecture	PCL	Random microfibers	Increasing spinning time	Supported differentiation rather than proliferation of melanocytes.	Stem cells of human hair follicle outer root sheath	[152]
Bioactivity	GelMA	Random nanofibers	Increasing spinning time	Supported endothelial cell and dermal ﬁbroblast adhesion, proliferation, and migration; accelerates skin wound healing in vivo.	Human dermal ﬁbroblasts and HUVECs	[155b]
	SF	Random nanofibers	Template‐assisted electrospinning	Higher proliferation of fibroblasts in the deep layer and higher differentiation of keratinocytes in the superficial layer of an artificial bilayer skin.	Human skin keratinocytes and human dermal fibroblast	[29]
	SF/PCL	Random nanofibers	Template‐assisted electrospinning	Increased in vivo wound healing rate and collagen deposition.	NIH3T3 fibroblasts	[28b]
	SF/decellularized human AM	Random nanofibers	Layer‐by‐layer electrospinning	Reduced hypertrophic scar formation in vivo.	Human adipose tissue‐derived MSCs	[181a]
Drug release and bioactivity	Keratin−fibrin−gelatin/poly(3‐hydroxybutyric acid)/gelatin/curcumin/mupirocin	Random nanofibers	Layer‐by‐layer electrospinning	Increased collagen deposition and granulation tissue formation; promoted gaseous exchange and absorption of exudates	Murine NIH3T3 and human HaCaT keratinocytes cell lines	[141]
	PLA/CA/thymoquinone	Random nanofibers	Increasing spinning time	Prevented common clinical infections and accelerated the rate of epithelialization; promoted the formation of granulation tissue.	Mouse fibroblast cells	[189]
Antibacterial material	PCL/AgNPs	Random nanofibers	Increasing spinning time	Control of dermal bacterial infections.	Cell free	[147]
	PVDF/copolymer of polystyrene and poly(4‐vinylpyridine)	Random nanofibers	Increasing spinning time	Prevented the attachment of bacteria; faster wound recovery.	L929 fibroblast cells	[148]
Antibacterial material, and bioactivity	PCL/CS	Random nanofibers	Layer‐by‐layer electrospinning	Supported cellular adhesion, infiltration, and proliferation; accelerates maturation of granulation tissue; promotes angiogenesis.	Human foreskin fibroblast and keratinocyte cells	[190]
	PCL/QCSP	Random nanofibers	Increasing spinning time	Accelerated wound‐healing processes compared with commercial dressing (Tegaderm^TM^); allowed higher collagen deposition, granulation tissue thickness, and angiogenesis.	Red blood cells (RBC) and L929	[150]
	Alginate/ZnO	Random nanofibers	Increasing spinning time	Mechanical and water‐related properties are similar to those of human skin.	L929 fibroblasts and human keratinocyte HaCaT cell line	[191]

### 3.6 Other Tissue Regenerations

Though neural, cardiac, bone, vascularization, and skin regeneration have been intensely investigated, the unique properties of electrospinning have also been put to the test for repair of other tissues such as liver, kidney, and esophageal tissue.^[^ ^162^ ^]^

Liver diseases affect millions of people every year and due to the limited treatment options, donor livers are the golden standard of treating such diseases.^[^ ^163^ ^]^ However, lack of sufficient donors leaves space for alternative treatment solutions such as tissue engineering. In this regard, PLGA/type I collagen 3D nanofibrous scaffolds have been used to maintain primary hepatocyte functions, which showed increased albumin secretion, higher urea synthesis, higher cytochrome P450 2C9 (CYP2C9) enzyme activity, and elevated transcription of hepatocyte‐specific cytochrome P450 (CYP450) genes compared with in primary human hepatocytes cultured on unmodified PLGA scaffolds.^[162a]^ Likewise, Grant and coworkers fabricated electrospun scaffolds composed of decellularized human liver ECM (hLECM) and compared them with individual ECM components: laminin‐521, collagen I, and fibronectin. Compared with the individual ECM proteins, the electrospun scaffolds were found to maintain hepatocyte growth and albumin production and affect hepatic gene expression of THLE‐3 hepatocytes.^[163b]^ Furthermore, hepatocytes/fibroblasts coculture systems have been developed using fibronectin‐coated CS nanofiber scaffolds to culture and maintain long‐term liver function.^[162b]^ Likewise, drug‐induced hybrid PCL−ECM scaffolds were found to maintain both hepatocyte growth and function for 5 days in vitro,^[162c]^ where the hybrid scaffolds were conducted by culturing and decellularizing 5637 human urinary bladder epithelials on PCL scaffolds. Finally, heparan sulfate‐coated polyethersulfone (PES) scaffolds have been found to improve human endometrial stem cell differentiation into functional hepatocyte‐like cells.^[162d]^

Chronic kidney disease like liver diseases also affects millions of people worldwide, making it a major health problem.^[^ ^164^ ^]^ Besides organ transplantation, dialysis is the current treatment for people suffering from chronic kidney disease. However, this is a costly method and affects the quality of life of patients. A solution to this challenge may be found using electrospinning for tissue regeneration. Electrospun PLA scaffolds have been fabricated for kidney tissue engineering and found to sustain a multipopulation of kidney cells including aquaporin‐1‐positive proximal tubule cells, aquaporin‐2‐positive collecting duct cells, synaptopodin‐positive glomerular epithelial cells, and von Willebrand factor‐positive glomerular endothelial cells.^[162g]^ PCL scaffolds have also been fabricated for kidney proximal tubules with sufficient mechanical stability, rapid diffusibility, tight cellular monolayer formation, and prolonged construct viability, exhibiting superior properties over existing proximal tubule models with regard to implantation purposes and continuous blood clearance.^[^ ^165^ ^]^ Finally, electrospun PCL/poly(3‐hydroxybutyrate*‐co‐*3‐hydroxyvalerate) bioresorbable scaffolds have been developed and can be promising for tissue‐engineered urinary bladder augmentation.^[162i]^

Esophageal tissue regeneration has started to get attention in the world of tissue engineering, as several medical conditions, such as esophageal cancer, require surgical procedures resulting in esophageal defects. As the current methods of reconstructing the damaged esophageal tissue are highly invasive and potentially fatal, tissue engineering using electrospinning techniques is a potential way of improving esophageal regeneration.^[^ ^166^ ^]^ Zhuravleva et al.^[162h]^ developed an electrospun polyamide‐6‐based scaffold for esophageal tissue regeneration. Papio hamadryas esophagus was decellularized to determine the morphology and physical properties of its ECM. This inspired the design of a scaffold with excellent mechanical properties, 90% porosity that allowed cell adherence, and elongation of AD‐MSCs and bone marrow‐derived mesenchymal stromal cells (BMD‐MSCs). Alternatively, Wu et al. used electrohydrodynamic jetting (e‐jetting) to manufacture aligned PCL/pluronic F127 fibers with controlled pore sizes. Here they found that scaffolds containing 8% pluronic F127 exhibited a similar ultimate tensile strength as that of the native esophageal tissue. In addition, the PCL/pluronic F127 scaffolds were found to improve primary human esophageal fibroblast proliferation and expression of VEGF compared with pure PCL scaffolds, thus providing a promising approach for esophageal tissue regeneration.^[166a]^ Finally, Park and coworkers developed electrospun polyurethane nanofibers for esophageal reconstruction in rats. After 4 weeks of transplantation, kreatin13 immunostaining revealed that the nanofibrous scaffolds with and without preseeding of hAD‐MSCs were found to significantly increase the thickness of the regenerated esophageal epithelium compared with 3D‐printed PCL scaffolds.^[166b]^

**Table** **7** shows the studies highlighted in this section. Although there are comparatively few studies focusing on these tissue regeneration fields, electrospinning has demonstrated strong potential in these still underexplored applications. Future studies will hopefully take them one step closer to clinic.

**Table 7:** 3D electrospun scaffolds for other tissue regeneration

Applications	Polymer substrate	Scaffold structures	Fabrication techniques	Functionality	Cell type	Refs.
Liver tissue regeneration	PLGA/type I collagen	Random nanofibers	Wet electrospinning	Increased primary human hepatocyte synthetic activity.	Primary human hepatocytes	[162a]
	CS/fibronectin	Random nanofibers	Increasing spinning time	Enhanced cellular adhesion and spreading.	Primary rat hepatocytes	[162b]
	PCL/ECM	Random microfibers	Increasing spinning time	Created a niche microenvironment for hepatocytes, supporting in vivo phenotype and function.	Human HepG2 hepatocytes	[162c]
	PES with collagen, heparan sulfate (HS) or collagen/HS; galactosilated PES	Random nanofibers	Increasing spinning time	Increased cell survival, attachment, and function, cells show glycogen storage, α‐fetoprotein, and albumin secretion.	Human endometrial stem cells	[162d]
	PLLA/human liver ECM/Bornstein and Traub type I collagen, powder from human placenta, human‐recombinant laminin 521, and human plasma fibronectin	Random microfibers	Increasing spinning time	Maintained hepatocyte growth and albumin production. Affect hepatic gene expression	THLE‐3 hepatocytes	[163b]
Kidney tissue regeneration	PLA	Random microfibers	Template‐assisted electrospinning	Sustained a multipopulation of kidney cells with aquaporin‐1 (proximal tubules), aquaporin‐2 (collecting ducts), synaptopodin (glomerular epithelial), and von Willebrand factor (glomerular endothelial) cells.	Primary rat kidney cells	[162g]
	PCL/L‐3,4‐dihydroxyphenylalanine/collagen IV	Random nanofibers	Template‐assisted electrospinning	Sufficient mechanical stability, rapid diffusibility, tight cellular monolayer formation, and prolonged construct viability and functionality.	Murine‐induced renal tubular epithelial cells and human conditionally immortalized proximal tubule epithelial cells	[165]
	PCL/poly(3‐hydroxybutyrate*‐co‐*3‐hydroxyvalerate)	Random microfibers	Increasing spinning time	Promoted the formation of urothelium.	Cell free	[162i]
Esophageal tissue regeneration	Polyamide‐6	Random microfibers	Increasing spinning time	Supported cell adhesion and survival.	Human adipose‐ and bone marrow‐derived mesenchymal stromal cells	[162h]
	PCL/pluronic F127	Scaffolds with aligned fibers and controlled pore size	Electrospinning writing	Improved cell proliferation and expression of VEGF and exhibited similar ultimate tensile strength as native esophagus.	Primary human esophageal fibroblasts	[166a]
	Polyurethane	Random nanofibers	Increasing spinning time	Increased thickness of the regenerated esophageal epithelium.	hAD‐MSCs	[166b]

## 4 Conclusion

Electrospinning is a versatile technology that allows innovative advancement with great promise to fabricate 3D fibrous scaffolds. Electrospinning possesses the ease of combining other additive technologies to create scaffolds with optimal mechanical, biological, and physical properties. The electrospinning technology is rapidly advancing with organ‐shaped tailored collectors and 3D printing features.

Compared with the traditional, densely packed electrospun submicrometer fibers, loosely packed 3D electrospun scaffolds hold larger surface areas, tunable pore diameters, adjustable densities, and controllable shapes, which can promote cell infiltration and nutrients exchange. Such 3D electrospun scaffolds have been proven as superior biomaterials in various tissue regeneration fields, as summarized in our Review. However, the industrial application of the final 3D electrospun products is still in its initial stages. Although a wide range of biomaterials and bioactive components have been used in electrospinning with promising results and significant advances, the industrialscale production for the 3D electrospun scaffolds is still in its infancy, especially when the precise control of the physical architecture and biochemical functionalization at submicrometer scale is desired.

The 3D electrospun scaffolds are believed to hold great potential in many different areas of regenerative medicine; however, it is possible to further expand the research areas and accelerate their clinical applications. The future direction should address the new possibilities of large‐scale production with precise control over physical structure and biochemical composition.

## Conflict of Interest

The authors declare no conflict of interest.
